# Revisiting the Molecular Roadmap for Sugar Crops: Genome Reading, Trait Writing and Variety Redesigning

**DOI:** 10.1111/pbi.70683

**Published:** 2026-05-13

**Authors:** Peilin Wang, Qibin Wu, Wenzhi Wang, Prakash Lakshmanan, Yangrui Li, Khushi Muhammad, Yuguang Wang, Youxiong Que

**Affiliations:** ^1^ State Key Laboratory of Tropical Crop Breeding, Institute of Tropical Bioscience and Biotechnology, Sanya Research Institute Chinese Academy of Tropical Agricultural Sciences Sanya China; ^2^ Key Laboratory of Sugarcane Biology and Genetic Breeding, Ministry of Agriculture and Rural Affairs, College of Agriculture Fujian Agriculture and Forestry University Fuzhou China; ^3^ Key Laboratory of Sugarcane Biotechnology and Genetic Improvement (Guangxi), Ministry of Agriculture and Rural Affairs, Sugarcane Research Institute, Guangxi Academy of Agricultural Sciences Nanning China; ^4^ Department of Biotechnology & Genetic Engineering Hazara University Mansehra Pakistan; ^5^ Engineering Research Centre of Agricultural Microbiology Technology, Ministry of Education & School of Life Sciences Heilongjiang University Harbin China

**Keywords:** conventional breeding, designed breeding, genetic engineering, genetic transformation, sugar crops

## Abstract

Sugar crops, including but not limited to sugarcane, sugar beet, sweet sorghum and stevia, are major sources of sugar production in the world. However, conventional breeding approaches, limited by long breeding cycles, low efficiency and restricted capacity to improve complex traits in sugar crops, are increasingly insufficient to address the challenges posed by climate change and the demands of sustainable agriculture. This review systematically summarizes recent advances in biotechnology and molecular breeding that have transformed sugar crop improvement. Recently, high‐throughput sequencing technologies have generated extensive multi‐omics resources. Concurrently, numerous functional genes and genetic elements with substantial breeding potential have been identified and cloned, offering precise targets for the key agronomic traits in sugar crops. Marker‐assisted selection has been successfully implemented to enhance disease resistance, while genomic selection has demonstrated well for the evaluation and selection of complex quantitative traits. Importantly, genetic transformation systems have enabled precise manipulation of target genes and facilitated the creation of novel germplasm. In the future, the integration of multi‐omics data, artificial intelligence, high‐throughput phenotyping and precision genome editing into an intelligent breeding framework will be essential for achieving breeding by design and developing climate‐adaptive and smart cultivars. Ultimately, these technological innovations will expand the role of sugar crops beyond traditional sugar production, positioning them as a central platform for sustainable biomanufacturing and providing critical support for global sugar security, energy transition and the development of the bioeconomy.

## 1 Introduction

Sugar crops form a foundational pillar of the global agricultural and industrial economy. Led by sugarcane (*Saccharum* spp.), sugar beet ( *Beta vulgaris* L.) and sweet sorghum ( *Sorghum bicolor* L.), these crops collectively contribute approximately 80% of global sugar (sucrose) production and serve as essential raw materials for the food industry (Rizvi et al. 2021; Lima and Beacorn 2022; FAO 2023; FAOSTAT 2024). Beyond their role in food production, sugarcane and sweet sorghum, owing to their high biomass yields and abundant fermentable sugars, have emerged as among the most important non‐grain feedstocks for first‐generation biofuels, particularly ethanol. As such, they constitute critical components of national energy‐security strategies in major producing countries, including Brazil, the United States, India and China (Budeguer et al. 2021). Meanwhile, *Stevia rebaudiana* , valued for its steviol glycosides, has experienced rapidly increasing demand as a source of natural zero‐calorie sweeteners, driven by global shifts toward healthier dietary preferences (Ceunen and Geuns 2013). In addition, regionally important species such as sugar palm ( *Borassus flabellifer* ) and sugar maple ( *Acer saccharum* ) supply culturally and economically significant sugar products, including palm sugar and maple syrup, in South Asia and North America, respectively (Mirinal 2023). Collectively, sugar crops generate substantial global trade value and employment opportunities, while their production systems and processing by‐products provide considerable potential for biorefining, animal feed and biobased materials, positioning these crops at the centre of the modern circular bioeconomy (Karp and Shield 2008; Rabelo et al. 2011).

Over the past century, conventional breeding strategies based on hybridization and population selection have delivered major gains in yield and quality across sugar crops. In sugarcane, for example, breeding programmes successfully combined the high sucrose content of the ‘noble’ cane *S. officinarum* with the strong stress tolerance of its wild relative *S. spontaneum* through wide hybridization and recurrent selection, enabling repeated cycles of cultivar replacement and productivity improvement (Piperidis and D'Hont 2020; Lu et al. 2024). In sugar beet, the development and exploitation of cytoplasmic male sterility systems facilitated the large‐scale commercial production of hybrids, leading to substantial increases in both root yield and sugar content (Dohm et al. 2014; Zhong et al. 2025). Despite these achievements, the limitations of conventional breeding have become increasingly apparent as production systems demand higher overall performance and face escalating pressures from climate change (Que, Wu, et al. 2014). A primary constraint lies in the extraordinarily complex genetic architecture of sugarcane, which is characterized by high heterozygosity and extreme polyploidy with numerous chromosomes. This complexity greatly hampers the genetic dissection of key quantitative traits and reduces the efficiency of identifying and fixing favourable alleles through traditional selection (Zhang, Qi, et al. 2025; Garsmeur et al. 2025; Wang, Pan, et al. 2025). Moreover, breeding cycles across sugar crops are typically prolonged—often spanning 10–12 years in sugarcane—and depend heavily on large‐scale, long‐term field phenotyping, requiring substantial investments of time, labour and resources (Jackson 2005; Wickramasinghe et al. 2024; Lu et al. 2024). These challenges are further compounded by increasing threats from biotic stresses, such as sugarcane brown rust caused by *Puccinia melanocephala* and sugar beet *rhizomania* caused by beet necrotic yellow vein virus, as well as from abiotic stresses including drought, salinity or alkalinity and extreme temperatures (Raboin et al. 2006). The impacts of these stresses are intensified by climate change and the continued reduction of available arable land (McGrath and Panella 2018). For emerging or minor sugar crops, such as stevia and sugar maple, the absence of well‐established, systematic breeding programmes has resulted in even slower genetic improvement and limited varietal advancement (Prakash and Chaturvedula 2016). Together, these factors severely constrain the speed, precision and adaptability of sugar‐crop breeding in response to future agricultural and environmental challenges.

To address the inherent constraints of conventional breeding, the integration of modern biotechnology with molecular breeding strategies has become an inevitable and transformative direction for sugar‐crop improvement. Omics‐based technologies, encompassing genomics, bioinformatics and high‐throughput phenomics, provide unprecedented opportunities to dissect the molecular basis of complex traits such as sugar accumulation, disease resistance, stress tolerance and growth and developmental regulation (Zhang et al. 2021; Kumar et al. 2024; Wang, Wang, Wang, et al. 2026). Marker‐assisted selection and genomic selection enable the rapid and accurate prescreening of target traits—particularly those that are difficult to phenotype reliably at early developmental stages—thereby allowing selection to be conducted at the seedling stage or in early generations, significantly enhancing selection efficiency and shortening breeding cycles (Mahadevaiah et al. 2021; Escamilla et al. 2025). At the same time, transgenic technologies and revolutionary genome‐editing approaches overcome reproductive barriers between species and permit site‐specific gene knockout, modification or precise introgression of elite exogenous genes. These approaches provide highly precise tools for generating novel germplasm with improved resistance to pests and diseases, enhanced stress tolerance and elevated sugar content (Mall et al. 2025; Wang, Gou, et al. 2025). Furthermore, the rapid development of synthetic biology and artificial intelligence–driven breeding has opened new avenues for rationally designing crop metabolic pathways and predicting phenotypic outcomes, accelerating the transition toward precision, efficiency and targeted genetic improvement (Zhang, Xu, et al. 2025). Collectively, the integrated deployment of these technologies is driving a fundamental shift in sugar‐crop breeding from experience‐based phenotypic selection to theory‐guided, genome‐informed design, representing a critical pathway toward precise, efficient and goal‐oriented breeding strategies.

This review aims to systematically synthesize and critically evaluate major advances achieved over the past decade in biotechnology and molecular breeding of both major and emerging sugar crops, including sugarcane, sugar beet, sweet sorghum, stevia, palms and sugar maple. We first summarize the development and current status of genomic and multi‐omics resources for each crop. We then examine representative case studies and the application of core technologies, including the discovery of genes controlling key agronomic traits, molecular marker development and marker‐assisted selection, genetic transformation and genome‐editing approaches. Finally, through a comparative analysis of both shared features and crop‐specific differences in technology deployment, we identify potential challenges and discuss how emerging frontiers—such as multi‐omics data integration, AI‐assisted breeding and synthetic biology—may collectively shape an integrated roadmap for future molecular design breeding in sugar crops.

## 2 Genomics and Multi‐Omics Resources for Sugar Crops

**FIGURE 1:**
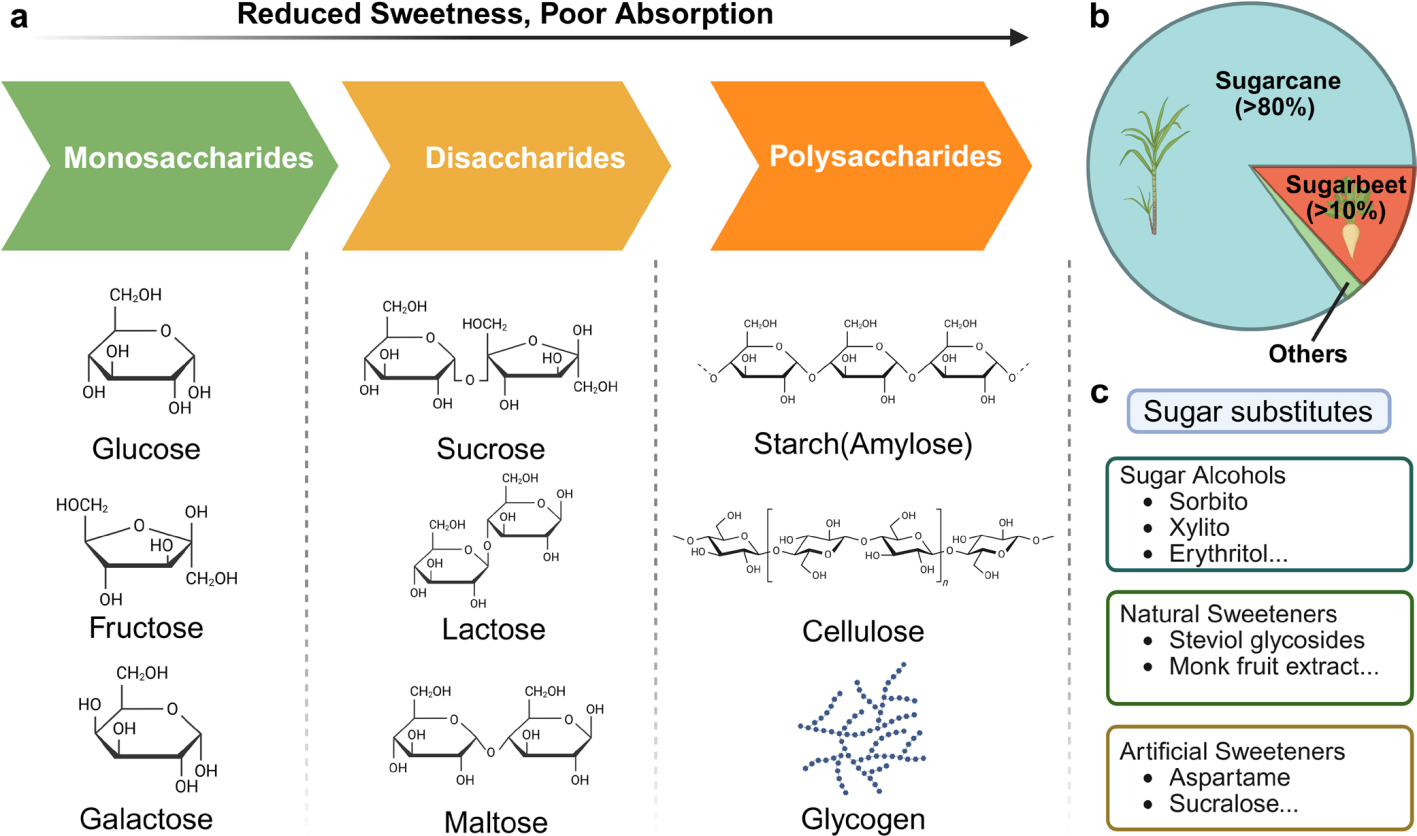
Types of sugars and dietary sugar sources. (a) Sugars and sugar‐related compounds classified by chemical structure into monosaccharides (e.g., glucose, fructose and galactose), disaccharides (e.g., sucrose, lactose and maltose), and polysaccharides (e.g., starch, cellulose and glycogen). For each sugar, only one representative chemical structure is depicted in this figure. (b) Proportional contribution of major dietary sources to global sugar consumption. (c) Classification of sugar substitutes (Sweeteners), including sugar alcohols, natural sweeteners and artificial sweeteners. Created with BioRender (http://www.BioRender.com).

Sugar crops have evolved from their botanical origins into indispensable components of global agriculture and the modern industrial economy. According to their chemical structures, sugars and sugar‐related compounds are commonly classified into monosaccharides (e.g., glucose, fructose and galactose), disaccharides (e.g., sucrose, lactose and maltose) and polysaccharides (e.g., starch, cellulose and glycogen). At a macroscopic level, a general trend can be observed in which kind of sweetness and, under specific conditions, absorption efficiency tend to decrease from monosaccharides to polysaccharides (Figure 1a). In industrial sugar production, sugarcane is the predominant crop, accounting for more than 80% of the global sugar supply, followed by sugar beet (Figure 1b; Table S1). In response to increasing health awareness and evolving consumer preferences, sugar substitutes (sweeteners) are being adopted more widely. These alternatives can be broadly assorted into sugar alcohols, natural sweeteners and artificial sweeteners (Figure 1c), of which the substitutes differ substantially in metabolic characteristics and application scenarios.

**FIGURE 2:**
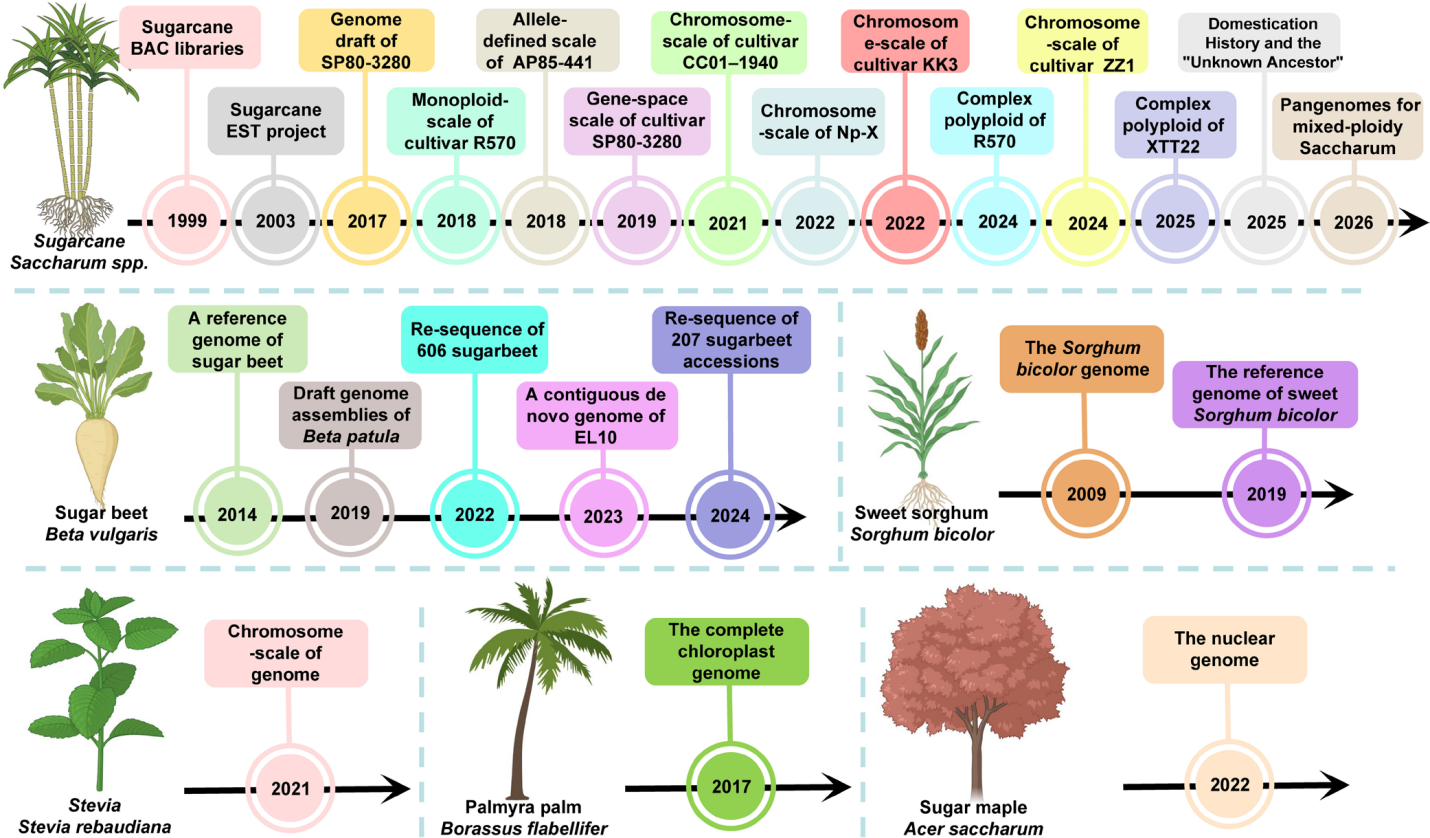
Timeline of genomic research advances in major sugar crops. Created with BioRender (http://www.BioRender.com).

Regarding genetics and breeding in sugar crops, the rapid advancement of high‐throughput sequencing technologies has fundamentally reshaped the research paradigms. The generation of high‐quality reference genomes, together with multidimensional datasets derived from transcriptomics, proteomics and metabolomics, has provided unprecedented global and high‐resolution resources for systematically dissecting the molecular basis of complex agronomic traits. Compared with many other crop species, high‐quality reference genomes are now available for several major sugar crops, including sugarcane, sugar beet and sweet sorghum. In contrast, genomic resources for minor or emerging sugar crops remain relatively limited, highlighting substantial disparities in resource development across species (Figure 2).

### 2.1 Progress in Genome Sequencing

Sugarcane is a highly polyploid (2n = 80–128) interspecific hybrid crop with an exceptionally complex genome, characterized by numerous chromosomes, extensive homology among homologous sequences and a high proportion of repetitive elements (~70%–80%). These features have long posed major challenges to whole‐genome sequencing and assembly. Consequently, early genomic studies relied primarily on fragmented approaches based on bacterial artificial chromosome (BAC) library sequencing and genetic map anchoring. The first sugarcane BAC library was reported in 1999 (Tomkins et al. 1999), followed in 2003 by the launch of the Sugarcane Expressed Sequence Tag (EST) initiative. This project generated extensive cDNA libraries and high‐quality full‐length transcripts that covered more than 90% of expressed genes, marking the formal beginning of sugarcane genomics research (Vettore et al. 2003).

Since 2017, significant progress has been achieved in sugarcane genome sequencing. In that year, a ~400 Mb draft genome of the cultivar SP80‐3280 was released (Riaño‐Pachón and Mattiello 2017). In 2018, the French National Institute for Agricultural Research (INRA) published a haplotype genome sequence of the modern cultivated sugarcane cultivar ‘R570’, representing approximately half of the monoploid sugarcane genome. This assembly integrated 4660 sugarcane BACs and captured gene‐rich regions syntenic with the closely related sorghum genome, thereby providing an essential framework for deciphering the genome organization of modern cultivated sugarcane (Garsmeur et al. 2018). In the same year, the first haploid chromosome‐level assembly of the homoeologous tetraploid (x = 8) wild sugarcane *Saccharum spontaneum* L. ‘AP85‐441’ was released. This work revealed a largely random distribution of *S. spontaneum* ancestry introgressed into modern sugarcane genomes (Zhang et al. 2018). Despite these advances, substantial gaps and incomplete assemblies persisted, continuing to limit genomic analyses and breeding applications.

Subsequent efforts progressively improved genome completeness and resolution. In 2019, an enhanced draft genome of the cultivated sugarcane ‘SP80‐3280’ provided new insights into the genetic basis of high biomass accumulation and revealed extensive biallelic and single‐nucleotide variation across the genome (Souza et al. 2019). In 2021, a haplotype draft genome of the cultivated sugarcane ‘CC01‐1940’ was reported, with a total assembled length of 903.2 Mb—substantially larger than the previously published 382 Mb ‘R570’ haplotype genome—and offering increased power for population genomics and trait‐mapping studies (Trujillo‐Montenegro et al. 2021). In 2022, the complete genome of the wild *S. spontaneum* accession ‘Np‐X’ was successfully resolved, clarifying the evolutionary origin of species and proposing a model for the formation of its complex genetic background (Zhang et al. 2022). In the same year, a partially chromosome‐scale genome analysis of the cultivated sugarcane ‘KK3’ revealed extensive recombination events and chimeric genomic structures (Shearman et al. 2022).

More recent breakthroughs have substantially advanced sugarcane genomics toward near‐complete resolution. In 2024, the genome of the cultivated sugarcane ‘R570’ was fully resolved, overcoming limitations of earlier physical and genetic maps and enabling the identification of putative causal genes underlying the single‐copy *Bru1* brown‐rust resistance locus (Healey et al. 2024). In parallel, a landmark study using the modern hybrid cultivar ‘Zhongzhe 1’ (ZZ1) integrated PacBio single‐molecule sequencing, high‐resolution Hi‐C–based physical mapping and Oligo‐FISH karyotyping to generate the most complete and highest‐quality genome of a modern hybrid cultivated sugarcane to date (Bao et al. 2024). In 2025, the genome of XTT22 was published, dissecting the complex genomic architecture of modern cultivated sugarcane, revealing subgenome‐specific expression changes following allopolyploidization, and, for the first time, identifying key loci associated with sugar‐related traits based on haplotype‐resolved genomes (Zhang, Qi, et al. 2025). Furthermore, whole‐genome sequencing combined with an innovative repeat k‐mer–based analytical framework enabled the first comprehensive reconstruction of the domestication history of cultivated sugarcane and uncovered a previously unknown wild ancestor that contributed genetic material to modern cultivars (Garsmeur et al. 2025). This discovery provides critical insights into sugarcane evolution and domestication and offers valuable guidance for mining novel genetic resources for future breeding (Zhang, Luo, et al. 2025). Most recently, a study introduced multiscale graph‐based pangenomes for mixed‐ploidy *Saccharum*, delivering the first pangenome graph framework explicitly designed for highly complex polyploid crops and enabling reference‐bias‐resistant genomic and multi‐omics dissection across diverse sugarcane genomes (Huang et al. 2026). Collectively, these advances provide a genomic blueprint for understanding sugarcane evolution and domestication and importantly, enable high‐resolution, genome‐wide identification of single‐nucleotide polymorphisms, structural variants and haplotype‐specific patterns linked to key agronomic traits, thereby helping to overcome breeding constraints imposed by extreme polyploid complexity.

In contrast to sugarcane, sugar beet ( *Beta vulgaris* L.) is a diploid species (2n = 18) with a genome size of approximately 758 Mb and a repetitive content of ~42%, making its genome substantially less complex and more amenable to sequencing. In 2014, the first sugar beet reference genome (~567 Mb) was published, marking the transition of sugar beet breeding into the genomics era (Dohm et al. 2014). Genome annotation identified approximately 27 000 protein‐coding genes and successfully localized the key bolting and flowering regulator *BvFL1*, as well as mitochondrial genomic regions associated with cytoplasmic male sterility. Subsequently, resequencing‐based population genomic studies expanded rapidly. In 2019, draft genome assemblies of *Beta patula* , a critically endangered wild beet endemic to the Madeira archipelago, and *Beta vulgaris ssp. maritima* (sea beet) were reported, comprising 25 127 and 27 662 genes, respectively (Rodríguez Del Río et al. 2019). In 2022, sequencing and analysis of 606 beet genomes, including sugar beet, sea beet, *B. vadanensis*, *B. macrocarpa* and *B. patula* , identified Greek populations as the closest wild relatives of sugar beet and suggested a domestication origin in this region (Sandell et al. 2022). More recently, a highly contiguous assembly of the inbred sugar beet line ‘EL10’ ( *B. vulgaris ssp. vulgaris* ) was produced, with the EL10.1 genome spanning 540 Mb and containing 24 255 gene models (McGrath et al. 2023). Resequencing of 207 accessions, combined with genome‐wide association studies and RNA sequencing, identified candidate genes regulating biomass and sugar accumulation, including the pleiotropic gene *UDP‐glucose 4‐epimerase*, which mediates a genetic trade‐off between sugar accumulation and cell wall biosynthesis (Wang, Yue, et al. 2024). In parallel, sugar beet genomics has expanded toward epigenomic analyses, with DNA methylation and histone modification profiling revealing regulatory mechanisms underlying bolting and developmental timing. Owing to its relatively compact genome, sugar beet has become one of the most advanced sugar crops for molecular breeding, with its reference genome widely applied in molecular marker development, QTL mapping, and gene cloning.

Sweet sorghum ( *Sorghum bicolor* L.) is another diploid species (2n = 20) with a genome size of approximately 730 Mb and exhibits high collinearity with other poaceae crops such as maize and rice. In addition to its importance as a sugar and biomass crop, sweet sorghum has also served as a model system for studying C4 photosynthesis and sugar transport. Its reference genome was first released in 2009 and annotated with 34 496 protein‐coding genes (Paterson et al. 2009). Subsequent improvements generated lineage‐resolved reference resources, enabling detailed structural and functional comparisons between sweet and grain sorghum types (Cooper et al. 2019). Comparative genomic analyses further revealed that sweet sorghum and sugarcane share an ancient whole‐genome duplication event, but differ in copy‐number expansion and expression divergence of key sugar‐metabolism gene families, including sucrose transporters of the SUT family and vacuolar invertases, providing insights into the divergence of their primary sugar‐storage organs (McCormick et al. 2018).

Beyond genomics, understanding the domestication history of sweet sorghum provides critical insights into the genetic basis of its high sugar content. Sorghum, the fifth most important cereal crop worldwide, originated in Africa with domestication dating back to approximately 5000–6000 years ago (Ge et al. 2022; Venkateswaran et al. 2019). Sweet sorghum represents a distinct type selected for juicy, sugar‐rich stems. During domestication and subsequent improvement, human selection mainly targeted multiple key genes governing domestication syndrome traits, including *Sh1* and SpWRKY (shattering), *SbTB1* (tiller number), *Dw* series (plant height) and *Dry* (stem juiciness) (Ge et al. 2022; Zhang et al. 2022). From Africa, sorghum spread globally through trade routes, reaching South Asia, the Middle East and eventually the Americas, with subsequent introduction to the United States in the 1850s and centuries of cultivation in China (Venkateswaran et al. 2019). Comparative genomic analyses further revealed that sweet sorghum and sugarcane share an ancient whole‐genome duplication event, but differ in copy‐number expansion and expression divergence of key sugar‐metabolism gene families, including sucrose transporters of the SUT family and vacuolar invertases, implying the divergence of their primary sugar‐storage organs (McCormick et al. 2018).

Genomic resources for other sugar crops are also expanding. The stevia genome has been resolved to chromosome scale, clarifying the genetic basis of steviol glycoside biosynthesis and enabling the cloning and functional characterization of key *UDP‐glycosyltransferase* genes involved in this pathway (Xu et al. 2021). As for sugar palm and sugar maple, genome sequencing efforts have also been initiated, including the chloroplast genome of Palmyra palm ( *Borassus flabellifer* ) and a nuclear genome assembly of sugar maple ( *Acer saccharum* ) (Sakulsathaporn et al. 2017; McEvoy et al. 2022). These emerging resources provide new opportunities for comparative genomics to identify conserved regulatory networks controlling sugar accumulation and species‐specific adaptive mechanisms.

Comparative analyses indicate that sugarcane and sweet sorghum share high sequence homology in key sugar‐metabolism genes, including *SUT* and *sucrose synthase (SuSy)*. In sugarcane, polyploidization has driven extensive copy‐number expansion of these genes, which may underlie its unique sucrose accumulation capacity. In contrast, while sweet sorghum and sugar beet exhibit greater overall genomic divergence, core genes involved in sucrose synthesis and transport remain functionally conserved, reflecting convergent evolutionary trajectories toward sugar accumulation across distinct sugar crops. In stevia, the availability of a reference genome has enabled identification of key enzymes in steviol glycoside biosynthesis, such as UGT74G1 and UGT85C2 (Richman et al. 2005). Together, the growing repertoire of genomic resources across sugar crops sets up a foundation for cross‐species functional validation, comparative analyses and the transfer of molecular breeding technologies.

### 2.2 Advances in Transcriptomics, Proteomics and Metabolomics

Single‐omics approaches are often insufficient to fully resolve the molecular mechanisms underlying complex traits in sugar crops. Integrated analyses combining transcriptomics, proteomics and metabolomics offer a continuous, multilevel perspective spanning gene expression, protein function and metabolite accumulation. Such coordinated multi‐omics strategies provide multidimensional evidence for identifying key genes and regulatory networks associated with important agronomic traits. By integrating time‐series and tissue‐specific transcriptomic and metabolomic data, researchers have resolved, at high spatial and temporal resolution, the dynamic regulatory networks controlling sucrose flux along the source–sink–flow continuum in sugarcane and sweet sorghum.

**FIGURE 3:**
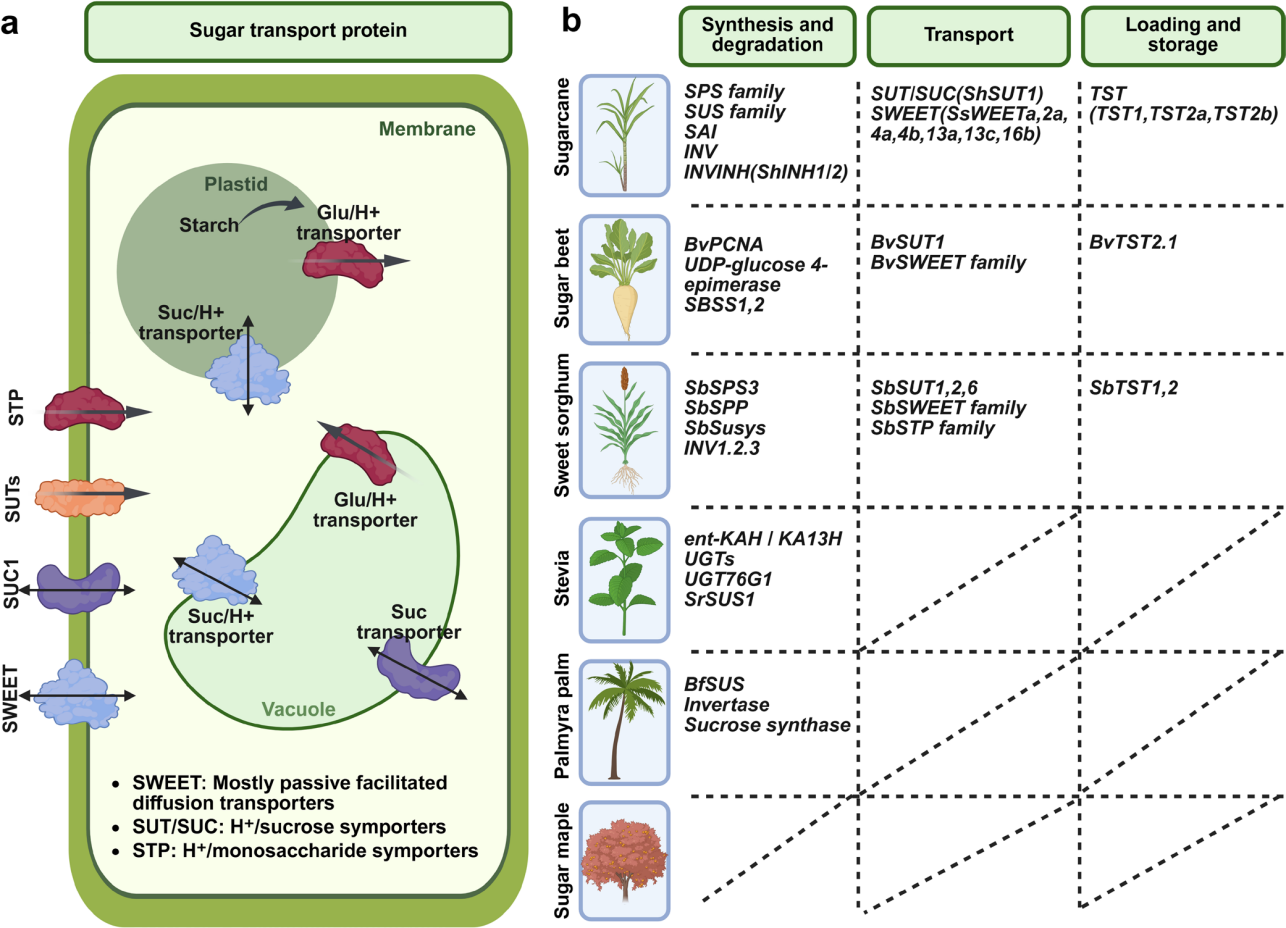
Action mechanisms of sugar transporters and genes associated with sugar metabolism. (a) Schematic representation of the major classes of sugar transporters and their functional roles in sugar transport. (b) Key genes involved in sugar synthesis and degradation, transport, phloem loading and storage in major sugar crops. A slash indicates that no relevant report is currently available. Created with BioRender (http://www.BioRender.com).

In studies of sugar metabolism, sugar transporters are commonly classified into three major groups: *SWEETs*, *SUTs*/*SUCs* and monosaccharide transporters (*MSTs*). In sugarcane, members of the *SWEET*, *SUT* and *STP* families are critical in sugar transport (Zhang et al. 2021) (Figure 3). Transcriptomic analyses further indicate that sucrose synthase genes *Sus1* and *Sus2* and sucrose‐phosphate synthase genes (SPS) *SPS1* and *SPS2* are strongly upregulated from the elongation stage to the maturation stage, whereas sucrose transporter genes *SUT1* and *SUT4* exhibit high, phloem‐specific expression. Together, these patterns reveal a coordinated temporal programme underlying sucrose synthesis, transport and accumulation (Schäfer et al. 2004; Ma et al. 2020; Wang, Li, et al. 2022; Zhao et al. 2026). Functional studies support a central role for sucrose synthase (*SuSy*) in sink tissues of sugarcane internodes and demonstrate a division of labour among different *SUTs* during phloem loading and unloading (Yuan et al. 2022).

In sugar beet, root development is characterized by a transition from an early pre‐storage phase—marked by rapid biomass accumulation but a low sucrose‐to‐glucose ratio—to a later phase of intensive sucrose storage. This developmental switch is accompanied by distinct changes in carbon partitioning and root fresh‐weight gain (Giaquinta 1979). At the sink‐tissue level, the extent and mode of sucrose hydrolysis directly influence the maintenance of high sucrose concentrations. Comparative analyses of sucrose‐metabolizing enzymes in taproots and fibrous roots revealed marked differences in invertase and sucrose synthase activities, suggesting spatial separation and independent regulation of sucrose cleavage pathways within sink tissues (Silvius and Snyder 1979). Followed by vacuolar sequestration, high sucrose accumulation in sugar beet also depends on efficient cellular uptake. Using a root‐disc system, active sucrose uptake and compartmentation in sink cells, with kinetic properties demonstrating a regulatory role for transmembrane transport processes (Saftner et al. 1983). Subsequent identification of a sucrose carrier in immature taproots shifted attention toward the molecular identity of uptake systems linking phloem unloading with cellular import (Lemoine et al. 1988). Ultimately, advances in membrane proteomics and electrophysiology led to the identification and functional validation of the tonoplast sucrose transporter *BvTST2.1*, whose proton‐coupled transport mechanism mechanistically explained the efficient accumulation of vacuolar sucrose in sugar beet taproots (Jung et al. 2015).

Source strength is another key determinant of sugar accumulation, relying on efficient phloem loading and sucrose export from source leaves. In sugar beet leaves, both transcriptional regulation and transport activity of the phloem sucrose symporter *BvSUT1* are negatively regulated by sucrose concentration, reflecting a feedback mechanism that adjusts carbon export to internal metabolic status (Vaughn et al. 2002). Further work demonstrated that protein phosphorylation plays a central role in sucrose‐mediated transcriptional regulation of *BvSUT1*, suggesting a signalling pathway that links sucrose sensing to transporter gene expression (Ransom‐Hodgkins et al. 2003). Beyond transport, the activity and developmental regulation of sucrose biosynthetic enzymes also shape source capacity. Comparative analyses in sugar beet leaves showed that sucrose‐phosphate synthase activity in the synthetic direction is markedly higher than that of sucrose synthase and invertases, varying with diurnal light cycles and differing systematically between young and mature leaves (Pavlinova et al. 2002).

In *S. rebaudiana* , integrated transcriptomic analyses using weighted gene co‐expression network analysis identified genes strongly correlated with the accumulation of both steviol glycosides and phenolic compounds across multiple genotypes. The co‐occurrence of *UDP‐glycosyltransferases* (*UGT76G1*, *UGT76H1*, *UGT85C1* and *UGT91A1*), enzymes of the phenylpropanoid pathway and 21 transcription factors—including *SCL3*, *WRKY11* and *MYB111*—indicates an extensive and synergistic regulatory network enhancing the biosynthesis of these compounds in stevia leaves (Simoni et al. 2024).

Transcriptomic approaches have also been widely applied to dissect stress‐response mechanisms in sugar crops. In sugarcane, transcriptome sequencing identified a chitinase gene involved in plant–pathogen interactions (Que, Su, et al. 2014). Following the infection by the brown rust pathogen, the expression of disease‐resistance–related genes, including NBS‐LRR and pathogenesis‐related protein genes, as well as genes involved in the salicylic acid signalling pathway, increases sharply (Gao et al. 2021). Overexpression of *ScCAX2* and *ScCAX3* has been shown to increase susceptibility to pathogen infection, suggesting a negative regulatory role in disease resistance and highlighting potential targets for resistance breeding (Wu et al. 2024). In sugar beet, salt stress induces strong transcriptional responses mediated by *BvbZIP* transcription factors, which influence abscisic acid signalling pathways. Salt stress also alters the abundance of 14–3‐3 proteins, significantly affecting cellular signalling and stress‐response processes (Skorupa et al. 2016; Yu et al. 2016; Wang, Stevanato, et al. 2019). Additional transcriptomic analyses revealed that enhanced activities of ascorbate peroxidase and glutathione S‐transferase improve reactive oxygen species scavenging under salt stress (Dong, Liu, et al. 2025). A meta‐transcriptomic survey further uncovered the widespread coexistence of viruses in field‐grown sugar beet exhibiting virus‐like disease symptoms in the United States (Chinnadurai et al. 2024). More recently, single‐cell transcriptomics has enabled cell type–specific dissection of sugarcane responses to smut disease, opening new avenues for high‐resolution analysis of cellular regulatory mechanisms underlying stress responses (Zang, Wu, et al. 2025).

Proteomics, particularly iTRAQ‐ and TMT‐based quantitative approaches, bridges the gap between transcriptomic profiles and phenotypic outcomes by characterizing protein abundance, post‐translational modifications and protein–protein interactions. In sugarcane, iTRAQ‐based analyses comparing early‐ and late‐maturing cultivars identified 3337 proteins and revealed 47 differentially expressed proteins enriched in pathways related to sugar metabolism, photosynthesis and stress responses, with fructose‐bisphosphate aldolase and protein disulphide isomerase highlighted as key regulators of sucrose synthesis (Ou et al. 2026). During smut infection, comparative proteomic analyses showed that differentially expressed proteins were closely associated with resistance responses, including β‐1,3‐glucanase, peroxidase, pathogenesis‐related protein 1, endo‐1,4‐β‐xylanase, heat shock proteins and lectins (Su, Xu, Wang, et al. 2016). Reanalysis of proteomic datasets further identified 507 differentially abundant proteins, including 49 involved in sugar metabolism–related processes such as photosynthesis, carbon fixation and starch and sucrose metabolism (Li et al. 2022). Protein–protein interaction analyses indicated that abscisic acid signalling mediated by protein phosphatase 2Cs is a dominant regulatory pathway, while late embryogenesis abundant proteins act as hub regulators of cold‐stress adaptation. MicroRNAs and cell wall–related genes were also implicated in cold‐response regulation, revealing multilayered transcriptional and post‐transcriptional control mechanisms (Huang et al. 2021). Additionally, proteome‐wide studies have established reference proteome maps for sugarcane stem tissues and identified proteins responsive to drought stress (Amalraj et al. 2010; Salvato et al. 2019). In sugar beet, proteomic analyses revealed reversible protein expression changes associated with seed priming and aging (Catusse et al. 2011), as well as extensive proteomic remodelling during infection by beet necrotic yellow vein virus (Webb et al. 2014). Subsequent comparative proteomics identified a phenylcoumaran benzylic ether reductase encoded by *Pyrc5* that is associated with disease resistance (Hejri et al. 2021).

Metabolomics, based on GC–MS and LC–MS platforms, is a direct readout of physiological status by profiling metabolite composition and abundance. In sugarcane, LC–MS/MS analyses identified diverse metabolites in stem tissues, including alkaloids and non‐alkaloid compounds with potential medicinal value (Rao et al. 2022). Expanded metabolomic surveys detected hundreds of metabolites across multiple classes, with flavonoids representing a major fraction. Integrated network analyses identified hub genes and transcription factor families—such as Tify, NAC, MYB‐related, C2C2‐Dof, WRKY and bHLH—as key regulators of phenylpropanoid biosynthesis, flavone and flavonol pathways and starch and sucrose metabolism (Yuan et al. 2022). Metabolomic profiling of stems and leaves from multiple sugarcane cultivars further revealed that differential metabolites are enriched in flavonoid, phenylpropanoid and isoflavonoid biosynthesis pathways, as well as in starch and sucrose metabolism, which is a foundation for metabolic engineering strategies aimed at optimizing sugar accumulation (Lou et al. 2025). Under drought stress, sugarcane accumulates osmoprotectants such as proline and betaine (Yang et al. 2023), while disease‐resistance studies have identified characteristic metabolic signatures associated with smut resistance (Que, Pan, et al. 2014; Su, Wang, Xu, et al. 2016; Li, Wu, et al. 2024). In leaf scald disease caused by *Xanthomonas albilineans* , resistant sugarcane genotypes have distinct pathogen‐induced metabolic responses at specific time points, involving amino acids, phenolics, flavonoids, polyamines and phytohormone signalling pathways (Bini et al. 2023).

In sugar beet, integrated transcriptomic and metabolomic analyses revealed extensive reprogramming during root storage, with substantial proportions of expressed genes and detected metabolites. Notably, respiration‐related genes, including bidirectional sugar transporters *SWEET17*, were strongly correlated with respiration rate (Fugate et al. 2024). Under low‐temperature stress, genes associated with abscisic acid and gibberellin signalling, as well as lipid biosynthesis, were significantly upregulated (Liu et al. 2023). These stress‐associated metabolites and regulatory genes are promising metabolic markers for screening stress‐tolerant cultivars. Overall, integrated multi‐omics analyses combining transcriptomics, proteomics and metabolomics have enabled the construction of gene–protein–metabolite regulatory networks governing sugar metabolism and stress responses. Within the sugarcane sucrose‐accumulation network, for example, elevated *SPS* expression promotes sucrose accumulation by enhancing sucrose‐phosphate synthesis, while *SUT* expression regulates transmembrane sucrose transport. Such network‐level insights define explicit regulatory nodes that can be targeted through precise genome‐editing strategies (Ma et al. 2020; Zhao et al. 2026).

### 2.3 Bioinformatics and Database Development

As reported, the rapid expansion of multi‐omics datasets has driven the parallel development of specialized bioinformatics tools and databases, which now constitute the foundation for data integration, global sharing and collaborative research in sugar‐crop biology. Publicly accessible genome and gene‐expression repositories have substantially accelerated research progress by enabling efficient data reuse and cross‐study comparisons. To date, three dedicated databases have been developed specifically for sugarcane: the Sugarcane Genome Database (http://sugarcane.gxu.edu.cn/scdb/) (Chen, Feng, et al. 2024), the Sugarcane Genome Hub (SGH; https://sugarcane‐genome.cirad.fr/) and SugarcaneOmics (https://ngdc.cncb.ac.cn/scod/) (Luo et al. 2025). Among them, SugarcaneOmics represents an integrative multi‐omics platform that addresses limitations of traditional single‐omics databases by consolidating five core data modules across sugarcane and its close relatives, including genomes, transcriptomes, variomes, featured genes and germplasm resources. Its genome module aggregates chromosome‐scale assemblies from multiple species and provides comprehensive functional annotations together with pan‐genome query tools. In addition, this platform integrates breeding‐related information and basic statistics for 1096 sugarcane germplasm accessions, facilitating germplasm evaluation and selection.

The Sugarcane Genome Hub consolidates genome sequences, gene annotations, expression datasets and genetic maps from multiple sugarcane accessions, including *S. spontaneum* and *S. officinarum* . It offers user‐friendly functionalities such as gene search, sequence alignment and expression‐profile visualization, making it a central resource for comparative and functional genomics studies in sugarcane. For sugar beet, the *Beta vulgaris* Genome Database (BvGB; https://bvseq.boku.ac.at/) provides genomic datasets representing different ecotypes, along with quantitative trait locus information, molecular marker resources and a curated collection of cytoplasmic male sterility–related genes, thereby offering comprehensive data support for molecular breeding and genetic improvement (Sandell et al. 2022; Dohm et al. 2014). In sweet sorghum, the Sorghum Genome SNP Database (SorGSD; https://ngdc.cncb.ac.cn/sorgsd/) (Luo et al. 2016) integrates genomic, transcriptomic and metabolomic datasets and includes comparative genomics information relative to maize and rice, enabling functional inference and cross‐species knowledge transfer (Liu et al. 2021).

In addition to crop‐specific platforms, general‐purpose repositories such as GenBank, the European Nucleotide Archive and TAIR host extensive multi‐omics datasets for sugar crops and are essential infrastructure for data preservation, accessibility and cross‐species analyses. For other sugar crops, including stevia and sugar maple, genome and transcriptome datasets are primarily deposited in international repositories such as NCBI and EMBL‐EBI, where they are accessible through specific accession numbers and associated project records.

## 3 Gene Discovery and Functional Dissection of Key Traits

In recent years, the rapid expansion of genomic and multi‐omics resources has provided powerful tools for directly mapping genes underlying important agronomic traits and for elucidating their biological functions. Especially, genetic studies in sugar crops have advanced from coarse quantitative trait locus (QTL) mapping toward the cloning of causal genes, identification of functional allelic variation and dissection of the molecular regulatory networks controlling complex traits.

### 3.1 Genes Associated With Yield and Sugar Metabolism

Sugar accumulation is the core economic trait of sugar crops. It depends on a coordinated cascade of processes, including photosynthate synthesis in source tissues, long‐distance transport through the phloem and unloading, compartmentation and storage in sink organs. *Sucrose transporters (SUTs)* play central roles in phloem loading and unloading. They influence multiple physiological processes, including hormone biosynthesis, stress responses, photosynthesis and reproductive and fibre development (Chen, Miller, et al. 2024). As key mediators of transmembrane sucrose movement, *SUTs* control sucrose flux across cellular membranes (Deng et al. 2023). In sugarcane, *ShSUT1* is localized to the phloem and is primarily responsible for long‐distance sucrose transport, whereas *ShSUT4* and *ShSUT5* are highly expressed in sink tissues, particularly stems, where they participate in sucrose unloading and vacuolar storage. Importantly, in high‐sugar cultivars, expression levels of these *SUT* genes and/or specific allelic variants are significantly associated with sucrose content (Zhao et al. 2026; Zhang et al. 2021; Akbar et al. 2025). In sugar beet, the sucrose‐specific transporter *BvTST2.1*, which is expressed in both source leaves and roots, mediates vacuolar sucrose uptake in taproot cells and is essential for efficient sucrose storage in sink tissues (Jung et al. 2015; Nieberl et al. 2017).

**FIGURE 4:**
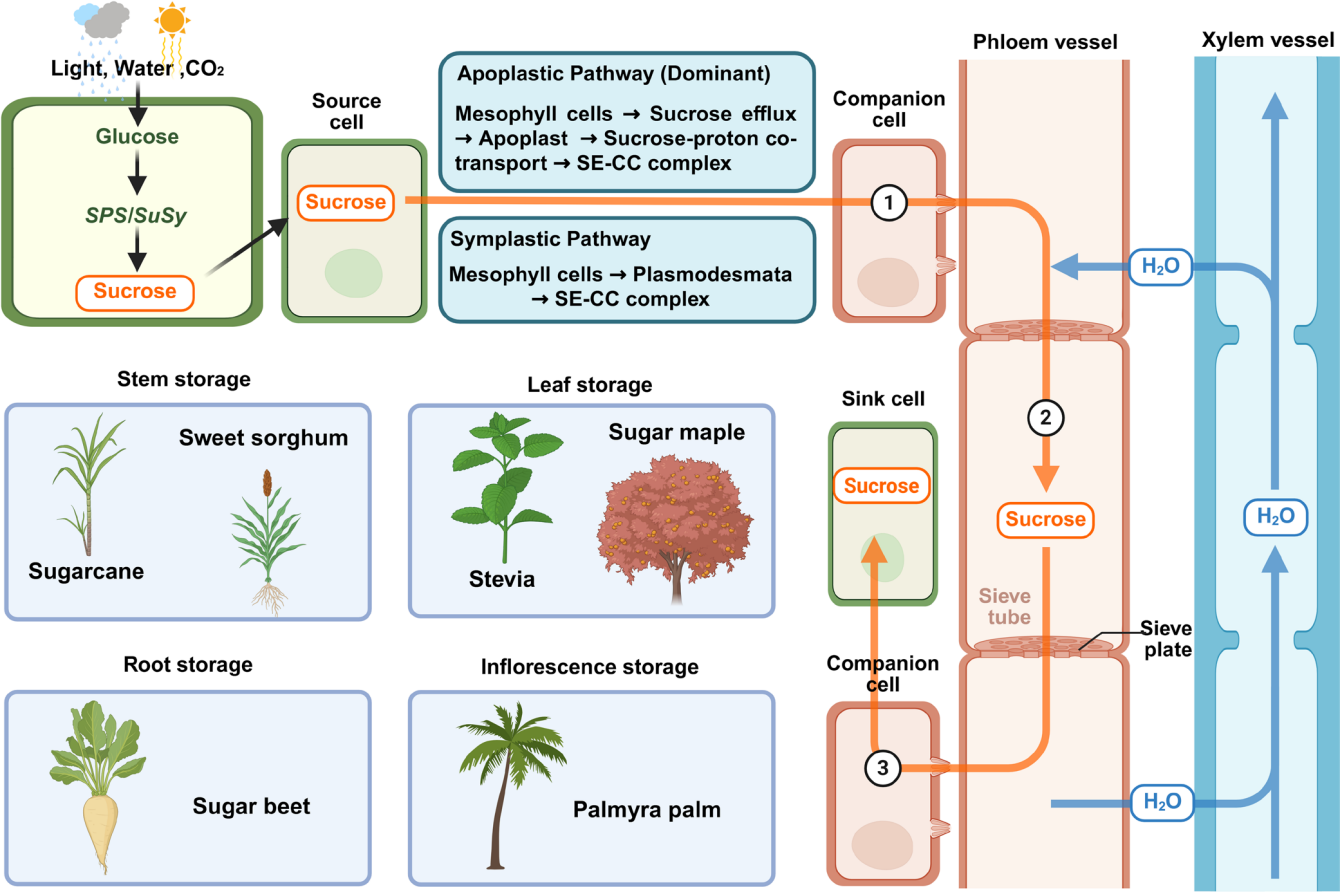
Major processes of ‘source–sink–flow’ in sugar transport and storage organs of major sugar crops. (1) Sucrose is synthesized in source tissues and loaded into phloem sieve tubes, leading to a reduction in water potential. (2) The resulting hydrostatic pressure gradient drives mass flow of phloem sap toward sink tissues. (3) Sucrose is unloaded from the phloem into sink cells, where it is utilized or stored.

Sucrose biosynthesis is catalyzed by *Sucrose‐phosphate synthase (SPS)* and *sucrose‐phosphate phosphatase (SPP)* (Figure 4). Among these enzymes, SPS functions as a key regulatory node and is reversibly activated or inactivated through protein phosphorylation in response to light conditions and osmotic stress (Huber and Huber 1996). In plants, SPS activity in both source and sink tissues contributes not only to sucrose resynthesis but also to the regulation of starch accumulation (Cheng and Chourey 1999), protein storage (Weber et al. 1997) and cellulose biosynthesis (Babb and Haigler 2001). In contrast, sucrose cleavage is *catalyzed by invertases (INV)* and *SuSy*. Invertases, which are important in carbon partitioning and developmental regulation, are classified into soluble acid invertase and neutral invertase (Wang, Zhao, et al. 2017), whereas SuSy contributes to the synthesis of cellulose, starch and proteins in sink tissues (Ruan 2014). In sugarcane, key sucrose‐metabolizing enzymes, including SPS, SuSy, soluble acid invertase (SAI) and neutral invertase (NI), are expressed in both leaves and stems (Kalwade and Devarumath 2014; Zhu et al. 1997). SPS activity is significantly higher in high‐sugar cultivars than in low‐sugar cultivars and is greater in mature internodes than in immature internodes (Verma et al. 2011). By contrast, SuSy and SAI activities are elevated in immature internodes. Together, high sucrose‐synthetic activity mediated by SPS and relatively low sucrose‐hydrolytic activity mediated by SAI are key determinants of sucrose accumulation in sugarcane. In addition, the activities of sucrose‐metabolizing enzymes are regulated at post‐transcriptional levels, and specific signalling pathways are thought to coordinate enzymatic switches that ultimately determine sucrose content (Papini‐Terzi et al. 2009).

Sugar transporters further regulate assimilate transport, compartmentation and storage and are generally categorized into monosaccharide transporters, *SUTs* and *SWEET* transporters. Two major sucrose‐loading pathways have been described: a symplastic pathway, which predominates in young sugarcane leaves, and an apoplastic pathway, which is more widely utilized during later developmental stages. Experimental evidence indicates that both the plasma membrane and the tonoplast of parenchyma cells mediate transmembrane sucrose influx and efflux (Moore 1995) (Figure 4). Despite their central role, relatively few sugar transporters have been functionally characterized in sugarcane. Apoplastic phloem loading and sucrose storage in parenchyma cells require the coordinated activity of multiple sugar transporters (Robinson‐Beers and Evert 1991; Wang et al. 2013). The sucrose transporter *ShSUT1*, localized in peripheral cells of vascular bundles, is proposed to function in sucrose retrieval during long‐distance phloem transport (Rae et al. 2005). Comparative genomic analyses have further characterized the structure and expression of *SUT* gene families in sugarcane (Zhang et al. 2016). *ShSUT1* and *ShSUT4* are abundantly expressed in both leaves and stems, *ShSUT2* shows comparable expression in both tissues, and *ShSUT5* and *ShSUT6* are predominantly expressed in leaves. In addition, *ShPST2a*, *ShPST2b* and *ShSUT4* are highly expressed in parenchyma cells, whereas *ShSUT1* shows preferential expression in vascular bundles (Casu et al. 2015). Collectively, these transporters form a coordinated network regulating sucrose accumulation in sugarcane.

In *S. rebaudiana* , leaf tissues are approximately 200–300 times sweeter than sucrose, a property attributable to steviol glycosides, a class of diterpenoid secondary metabolites dominated by *stevioside* and *rebaudioside A*. These compounds are synthesized through a dedicated steviol glycoside biosynthetic pathway. UDP‐glycosyltransferases play pivotal roles in the final steps of steviol glycoside biosynthesis, and the catalytic properties of enzymes such as UGT76G1 and UGT91D2 largely determine the accumulation of desirable components, particularly rebaudioside A (Brandle and Telmer 2007).

Final yield is ultimately determined by the size and metabolic activity of sink organs (Figure 4). The activities of cell wall invertase and vacuolar invertase influence sugar import into sink tissues and shape their metabolic status, while genes in phytohormone signalling pathways indirectly regulate sink strength by controlling cell division and expansion. During early taproot development, integrated profiling of enzyme activities, metabolites and phytohormone levels revealed significantly increased activities of multiple sugar‐related enzymes in sugar beet, including SuSy, phosphoglucose isomerase, phosphoglucomutase, phosphofructokinase, glucose‐6‐phosphate dehydrogenase and UDP‐glucose pyrophosphorylase, underscoring the coordinated metabolic reprogramming that underlies sink establishment and yield formation (Jammer et al. 2020).

### 3.2 Genes Associated With Biotic and Abiotic Stress Resistance/Tolerance

Extensive genetic analyses of major sugarcane diseases, including smut and rust, have identified multiple major‐ and minor‐effect quantitative trait loci, as well as functional genes operating through diverse defence mechanisms (Ling et al. 2022; Sun et al. 2024; Zou et al. 2024; Zang, Wang, et al. 2025; Zeng et al. 2025). Sugarcane smut, caused by *Sporisorium scitamineum*, represents one of the most destructive diseases affecting global sugarcane production (Que, Xu, et al. 2014; Xu et al. 2014; Chakraborty et al. 2024; Ling et al. 2025). Resistance to smut involves the coordinated activation of multiple defence pathways, among which genes associated with phenylpropanoid and lignin biosynthesis play prominent roles. Members of the *ScDIR* family (e.g., *ScDIR5*, *ScDIR7*, *ScDIR11* and *ScDIR40*), together with phenylalanine ammonia‐lyase, 4‐coumarate:CoA ligase and cinnamyl alcohol dehydrogenase, enhance cell‐wall lignification and strengthen mechanical barriers, thereby restricting pathogen invasion and spread (Su, Wang, Liu, et al. 2016; Singh et al. 2019).

Oxidative stress–related genes constitute another critical layer of defence. Genes encoding superoxide dismutase, catalase, ascorbate peroxidase and respiratory burst oxidase homologues mediate rapid oxidative bursts characterized by hydrogen peroxide accumulation, which in turn trigger hypersensitive responses and programmed cell death, limiting pathogen colonization (Hemetsberger et al. 2015; Peters et al. 2017; Wang, Que, and Zhang 2026). Disease‐resistance signalling components and transcription factors are also central regulators. WRKY family members (e.g., ScWRKY2), nucleotide‐binding site–leucine‐rich repeat (NBS‐LRR) genes, mitogen‐activated protein kinases and basic leucine zipper transcription factors integrate upstream perception signals and regulate downstream defence gene expression. These components activate salicylic acid– and jasmonic acid–dependent signalling pathways, enabling rapid pathogen recognition and effective defence responses (Djamei et al. 2011; Xia et al. 2020; Agisha et al. 2022; Sun et al. 2023; Wang, Wang, et al. 2024; Wang, Gou, et al. 2025; Wang, Zhang, Zou, et al. 2026). Pathogenesis‐related genes, including *PR1*, *PR2* (*β‐1,3‐glucanase*), *PR3* (*chitinase*) and *PR5*, directly inhibit pathogen growth and participate in cell‐wall remodelling and antimicrobial compound biosynthesis (Su, Wang, Xu, et al. 2016; Zang, Wu, et al. 2025; Zang, Wang, et al. 2025). Phytohormone signalling pathways are also indispensable for resistance. Genes involved in salicylic acid biosynthesis and signalling (e.g., *ICS* and *NPR1*), jasmonic acid signalling components (e.g., *COI1* and MYC2) and ethylene signalling genes (e.g., *EIN3*) coordinate hormonal cross‐talk to enhance systemic acquired resistance (Chen et al. 2001; Bhuiyan et al. 2021; Nagarajan et al. 2023). In addition, Ca^2+^/H^+^ exchangers regulate intracellular calcium homeostasis and four sugarcane genes (*ScCAX1–4*) have been implicated in smut resistance (Zhang et al. 2024). Finally, genes involved in flavonoid and phytoalexin biosynthesis, including chalcone synthase, chalcone isomerase, flavanone 3‐hydroxylase and dihydroflavonol 4‐reductase, contribute to the production of antimicrobial secondary metabolites which provide early‐stage defence against pathogen invasion (Ramaroson et al. 2022; Patil et al. 2024).

Map‐based cloning has enabled the isolation of several resistance genes encoding NBS‐LRR proteins. Among them, the sugarcane brown‐rust resistance gene *Bru1* has conferred durable resistance across multiple production regions (Asnaghi et al. 2004). Resistance to sugarcane mosaic virus is primarily mediated by resistance‐protein genes and host factors that interfere with viral replication. In sugar beet, the dominant resistance genes *Rz1* and *Rz2*, which confer resistance to beet necrotic yellow vein virus—the causal agent of rhizomania—have been cloned and shown to encode CC‐NBS‐LRR proteins (Gidner et al. 2005). In addition, cloning of the nematode‐resistance gene *Hs1pro‐1* has provided a precise molecular target for breeding resistance against the beet cyst nematode *Heterodera schachtii* (Thurau et al. 2003).

Abiotic stress tolerance, including resistance to drought, salinity and low temperature, is typically governed by complex, polygenic regulatory networks (Yang, Zhang, et al. 2017; Su et al. 2020; Wang, Zhai, et al. 2022). The abscisic acid signalling pathway serves as a central regulatory hub in these responses. Genes encoding core ABA signalling components, such as PYR/PYL receptors, PP2C phosphatases and SnRK2 kinases, play pivotal roles in stress adaptation (Zhang, Luo, et al. 2025). In sugarcane, overexpression of ScPYL61 enhances drought tolerance by reducing water loss and promoting stomatal closure through increased ABA sensitivity. Functional analyses revealed that ScPYL61 interacts with ScPP2C49 to form a ScPYL61*–*ScPP2C49*–*ScSnRK2s signalling module that mediates ABA‐dependent drought responses (Dong, Lu, et al. 2025). Under mild water deficit, both the activity and transcriptional abundance of Rubisco are altered (Zhang et al. 2013), while early drought stress reduces photosystem II efficiency and maximum quantum yield (Jangpromma et al. 2010; Cha‐um et al. 2012; Leanasawat et al. 2021).

Despite the activation of osmotic adjustment and the accumulation of compatible solutes, salinity stress severely constrains sugarcane productivity. Salinity‐induced reductions in photosynthetic efficiency, compromising yield and quality (Plaut et al. 2000; Akhtar et al. 2003; Tang et al. 2021), lead to decreased stem sucrose concentration. In sugar beet, an open reading frame encoding a *cysteine proteinase* inhibitor (*cystatin*) was isolated from the doubled haploid line M14 (*BvM14*). Arabidopsis plants overexpressing *BvM14‐cystatin* exhibited enhanced salt tolerance, demonstrating its functional role in salinity resistance (Wang et al. 2012). Waterlogging stress also poses a major threat to sugar beet production. Under waterlogging conditions, reactive oxygen species, hydrogen peroxide and superoxide levels increase in leaves, accompanied by elevated antioxidant enzyme activities and corresponding gene expression (Sha et al. 2024). Soil pH further exerts strong effects on sugar beet growth. Analyses across a wide pH range revealed that decreasing soil pH from 9.0 to 5.0 led to increased osmotic adjustment compounds, antioxidant enzyme activities and elemental contents in leaves and roots, whereas photosynthetic capacity and macronutrient contents declined (Wang, Dong, et al. 2022).

A wide range of stress‐responsive genes have been investigated in sugarcane, sugar beet and sweet sorghum, including genes encoding enzymes involved in osmoprotectant biosynthesis (e.g., *P5CS* and *BADH* for proline and betaine synthesis), reactive oxygen species–scavenging enzymes (superoxide dismutase, catalase and peroxidase), late embryogenesis abundant proteins, lipid transfer proteins and plasma membrane intrinsic proteins (Chagas et al. 2008; Chen et al. 2017; Geng et al. 2020; Jain et al. 2025). Their stress‐induced expression patterns have been extensively characterized and validated through transgenic approaches. Transcription factors such as DREB/CBF, NAC, MYB and WRKY function as upstream regulatory switches that coordinately activate suites of downstream stress‐responsive genes, making them particularly attractive targets for molecular breeding to improve broad‐spectrum resistance (Salvato et al. 2019; Li et al. 2026).

### 3.3 Genes Associated With Development and Agronomic Traits

To support mechanized production and adaptation across diverse agroecological zones, genetic improvement of agronomic traits such as plant architecture and phenology has become increasingly important. In sugarcane and sweet sorghum, studies on flowering regulation primarily aim either to promote flowering to facilitate hybridization or to suppress flowering to prevent diversion of assimilates from vegetative growth. In sugarcane, flowering control has a pronounced impact on yield (Pavani et al. 2023). Multiple homologues of FLOWERING LOCUS T (*ScFT1–ScFT13*) have been identified, and *ScFT3* has been functionally validated as a florigen controlling flowering induction (Wickramasinghe et al. 2024). In addition, overexpression of *ScGA20ox* increases gibberellin levels and accelerates vegetative growth (Wang, Li, et al. 2025). Manipulation of genes regulating tiller angle and tiller number, such as *TEOSINTE BRANCHED 1 (TB1)* homologues, offers opportunities to optimize canopy architecture by improving light interception and facilitating mechanized operations while enhancing resource‐use efficiency and stress adaptability (Aitken et al. 2008).

In biennial crops such as sugar beet, premature bolting and flowering can severely reduce root yield and sugar content. Several core flowering‐pathway genes, including *FLC*, *FT* and *SOC1*, have been identified and functionally characterized. Among them, *BvFL1* (a *FLOWERING LOCUS T homologue*) and *BvSOC1* (*SUPPRESSOR OF OVEREXPRESSION OF CONSTANS 1*) act as key repressors that control vernalization requirements. Allelic variation at these loci is directly associated with differences in flowering time among cultivars and has been widely adopted as molecular markers for breeding bolting‐resistant varieties (Pin et al. 2012). In addition, *BvBTC1* (*BOLTING TIME CONTROL 1*) functions as a central regulator of bolting, and mutations in this gene significantly delay the transition to flowering (Dally et al. 2014).

In sweet sorghum, biomass accumulation is closely linked to plant height and stem thickness. Three major dwarfing genes, *Dw1*, *Dw2* and *Dw3*, regulate plant height, with *Dw3* encoding an ATP‐binding cassette transporter that plays a critical role in stem elongation (Multani et al. 2003). Tiller development, which influences biomass partitioning, is regulated by TEOSINTE BRANCHED 1 (*SbTB1*) (Kebrom et al. 2006). In both sugarcane and sorghum, genes involved in strigolactone and auxin signalling pathways—including *D3*, *D10*, *D14*, *D27* and *TB1* homologues—have been shown to exert strong control over tiller number and architecture (Zheng et al. 2025). Targeted manipulation of these genes could facilitate the development of ideotypes suited for dense planting and mechanized harvesting, characterized by moderate tillering, reduced plant height and improved lodging resistance.

Beyond plant height and tillering, additional agronomic traits are increasingly being targeted. In sugarcane, genes associated with stem hardness, fibre content and leaf‐shedding are receiving growing attention due to their relevance for harvesting efficiency and processing quality. In sugar beet, breeding efforts also focus on storage‐root traits, including root shape—where conical roots favour efficient harvesting and washing—crown size, and resistance to mechanical damage, all of which are critical for yield stability and post‐harvest quality.

To provide an overview of the major genes functionally characterized in sugar crops, we summarize the key genes associated with yield/sugar metabolism, biotic/abiotic stress responses and developmental/agronomic traits for each species in Table S2.

## 4 Development of Molecular Markers and Marker‐Assisted Breeding

Toward genotype‐based selection, the development and application of molecular markers represent a fundamental shift in sugar‐crop breeding from phenotype‐based selection. This technological framework enables direct, rapid and accurate identification of target genes, substantially improving selection efficiency and breeding predictability. These advantages are particularly pronounced in early breeding generations and for traits that are difficult or costly to evaluate phenotypically, including recessive traits.

### 4.1 From SSRs to High‐Throughput SNPs and InDels

In sugar crops, the evolution of molecular marker systems has progressed from low‐throughput platforms to high‐throughput, genome‐wide approaches (Aitken 2022). Early‐generation simple sequence repeat (SSR, microsatellite) markers were widely adopted because of their high polymorphism, codominant inheritance and strong reproducibility. They played important roles in genetic map construction, cultivar identification and diversity analysis in sugarcane and sugar beet. However, both marker development and genotyping throughput for SSRs are inherently limited, constraining their utility in large‐scale breeding programmes.

A major technological transition has occurred with the widespread adoption of single‐nucleotide polymorphism (SNP) markers. SNPs, which are extremely abundant and densely distributed across plant genomes, are highly compatible with automated, high‐throughput genotyping platforms. With the availability of reference genomes and the continued reduction in sequencing costs, genome‐wide SNP discovery based on reduced‐representation sequencing methods—such as genotyping‐by‐sequencing (GBS) and RAD‐seq—or whole‐genome resequencing has become a routine strategy. In an early comparative study, SSR markers were used to genotype 35 cold‐tolerant sugarcane accessions. Genetic similarity coefficients derived from genomic SSRs ranged from 0.4138 to 0.8046 (mean 0.6251), whereas those based on expressed sequence tag SSRs ranged from 0.5145 to 0.7971 (mean 0.6651), with genomic SSRs showing superior discriminatory power (Liao et al. 2012). In another application, markers linked to *Bru1* and the brown‐rust resistance locus G1 were used to screen 164 sugarcane accessions in China. Only 14% of accessions carried the Bru1‐linked marker, 5.56% carried the *G1* locus and 3.66% carried both loci, indicating that most domestic cultivars lack brown‐rust resistance genes and it is a good scientific basis for targeted mining of resistance resources (Bowen et al. 2020).

In sugar beet, resequencing‐based approaches have enabled the rapid identification of millions of SNP markers, forming a foundation for high‐resolution genetic mapping and population analyses (Stevanato and Biscarini 2016). Despite its extreme genomic complexity, haplotype‐resolved genome assemblies now similarly support large‐scale SNP discovery and facilitate discrimination among alleles located on homoeologous chromosomes in sugarcane. In addition to SNPs, insertion/deletion (InDel) polymorphisms represent an important class of genomic variation and remain valuable for targeted genotyping, as they can be detected using simple and cost‐effective assays such as gel‐based methods. Recently, the development of high‐throughput SNP array platforms, including Illumina BeadChip systems, has further standardized and accelerated large‐scale genotyping in breeding populations. Beyond SSRs, SNPs and InDels, alternative marker systems have also been explored. Start codon–targeted (SCoT) markers, for example, have been applied to assess genetic diversity in sugarcane (Que, Pan, et al. 2014).

### 4.2 High‐Density Genetic Linkage Maps and QTL Mapping

The availability of large‐scale SNP datasets has enabled the construction of genetic linkage maps with unprecedented marker density, thereby greatly increasing the precision of quantitative trait locus (QTL) mapping for important agronomic traits. In sugarcane, QTL detection is particularly challenging due to its highly polyploid genome, which requires explicit consideration of allele dosage effects and interactions among homoeologous chromosomes. Nevertheless, the development of high‐density, haplotype‐based linkage maps has made it increasingly feasible to localize QTLs at the subgenome level.

Early progress in sugarcane QTL mapping was driven by methodological innovations that addressed the incompatibility between polyploid segregation patterns and conventional diploid mapping assumptions. Foundational studies demonstrated analytical strategies for linkage‐map construction and QTL detection under complex autopolyploid inheritance, providing a conceptual and methodological framework for subsequent genetic analyses of quantitative traits in sugarcane (Ming et al. 2001; Aitken et al. 2006). As marker technologies advanced, QTL studies transitioned from low‐density markers such as SSRs to high‐density SNP‐based platforms. Map‐saturation analyses using representative cultivars showed that increasing marker density substantially improves both QTL detection power and confidence‐interval resolution (Liu et al. 2016). In the high‐throughput era, genotyping‐by‐sequencing–based approaches have enabled the construction of single‐dose marker frameworks and facilitated large‐scale SNP‐based QTL mapping for yield‐related traits while explicitly accounting for key features of polyploid inheritance, including allele dosage and variant expression (Pastina et al. 2012).

Disease resistance represents a critical target for sugarcane improvement and, relative to yield‐ and quality‐related traits, is more amenable to forming a complete breeding pipeline from QTL discovery to validation and deployment. For rust diseases, high‐density genetic maps have enabled the identification of resistance QTLs for both brown rust and orange rust, establishing a QTL‐centred framework for genetic dissection and marker‐assisted selection (Asnaghi et al. 2004; Le Cunff et al. 2008; Balsalobre et al. 2017; Yang, Sood, et al. 2017). In the case of smut resistance, greater emphasis has been placed on integrating complementary methodologies and converting QTLs into deployable markers. By combining high‐density linkage mapping with bulked segregant RNA sequencing to detect resistance‐associated QTLs and converting key variants into kompetitive allele‐specific PCR (KASP) markers, a complete workflow has been established from QTL identification to practical molecular diagnostics (Yang et al. 2018; Gao et al. 2022). In recent years, multiple major‐ and minor‐effect QTLs controlling stalk yield, sugar content (including brix and sucrose concentration), fibre content and resistance to smut and rust have been mapped with increasing precision. In several cases, QTL intervals have been narrowed to physical regions containing only a limited number of candidate genes (Cheng et al. 2025; Fang et al. 2025; Wang, Pan, et al. 2025).

Genome‐wide association studies have further expanded the scope of genetic dissection in sugarcane. A genome‐wide resequencing analysis of 219 elite cultivated varieties identified approximately 6 million high‐quality SNPs and subsequent association analysis detected 2198 SNPs significantly associated with sucrose content, effective stalk number, plant height, stalk diameter, cane yield and sugar yield (Li, Chen, et al. 2024). For virus disease resistance, an integrated study using 285 F1 progeny derived from a cross between YT93‐159 (moderately resistant) and ROC22 (highly susceptible). It combined natural infection surveys across 11 environment samples from five ecologically distinct sites with phenotyping under three artificial inoculations involving mixed viruses (SCMV and SrMV). QTL mapping based on a high‐quality SNP linkage map identified candidate genes potentially underlying mosaic disease resistance (Lu et al. 2023). Sugarcane brown stripe disease, caused by *Helminthosporium stenospilum*, is another major foliar disease. Using single‐dose markers from the Axiom Sugarcane100K SNP array, high‐density linkage maps were constructed for the resistant cultivar YT93‐159 and the susceptible cultivar ROC22, leading to the detection of 32 QTLs associated with brown stripe disease resistance (Cheng et al. 2025). Similarly, an F_1_ population derived from YT93‐159 and ROC22 was phenotyped over six consecutive years for studies of leaf blight resistance. Extreme‐resistant and extreme‐susceptible bulks were constructed, and a polyBSA‐seq strategy identified four linkage markers associated with resistance, including three resistance‐linked and one susceptibility‐linked marker, with clear potential for deployment in breeding programmes (Wang, Ren, et al. 2022).

For complex agronomic traits, biparental QTL mapping in polyploid crops such as sugarcane offers the advantages of controlled genetic backgrounds and robust detection of segregating loci, but its mapping resolution and allelic diversity are inherently limited. In contrast, genome‐wide association studies leverage broader germplasm diversity to improve mapping resolution and capture a wider spectrum of allelic variation, although they require stringent correction for population structure and high marker density. Consequently, an integrated strategy combining both approaches is increasingly favoured: biparental QTL mapping is used to identify robust candidate loci, followed by GWAS to test consistency across diverse panels and refine QTL intervals, ultimately generating reproducible and deployable marker sets. Consistent with this framework, GWAS conducted in a Louisiana core germplasm collection identified marker–trait associations for yield components and sucrose‐related traits (Barreto et al. 2019; Fickett et al. 2019; Cortes et al. 2024).

As far as we know, the construction of high‐density SNP linkage maps and QTL mapping are generally more straightforward and efficient in sugar beet, sweet sorghum and stevia than in sugarcane. In sugar beet, QTLs controlling root yield, sugar content, impurity accumulation (e.g., potassium and sodium), bolting resistance and resistance to multiple diseases—including root diseases, Cercospora leaf spot and nematodes—have been finely mapped and several major‐effect loci have been converted into tightly linked or functional markers for marker‐assisted selection (Stich and Inghelandt 2018). In one study, an F2 population of 200 individuals derived from a cross between ‘JV34‐2’ and ‘2B023’ was analysed using sequence‐related amplified polymorphism and SSR markers, resulting in the identification of seven QTLs for storage‐root yield and ten QTLs for sugar content (Wang, Li, et al. 2017). In another study, an F1 population of 219 individuals derived from a cross between parents 3a and 3b was analysed using a high‐density genetic map, leading to the detection of 32 QTLs for four traits and the identification of thousands of candidate genes associated with root morphology, root weight and sugar content (Wang, Xu, et al. 2019). For disease resistance, QTL mapping using recombinant inbred lines and multi‐year field phenotyping dissected the genetic architecture of Cercospora leaf spot resistance and identified four stably expressed resistance QTLs (Taguchi et al. 2011). In sweet sorghum, high‐density linkage mapping has enabled extensive QTL identification for biomass, sugar content, flowering time and stress tolerance. Using a recombinant inbred line population derived from parents with contrasting plant height and sugar content, QTL mapping based on reduced‐representation sequencing identified multiple major and additive QTLs for plant height and sugar content, including loci showing significant genotype‐by‐environment interactions, thereby providing a genetic foundation for sorghum improvement (Shi et al. 2025). In stevia, an economically important perennial medicinal plant valued for its zero‐calorie sweeteners, molecular marker approaches have been applied to dissect the genetic basis of sweetness‐related traits. QTL analyses identified 53 QTL positions associated with rebaudioside A and stevioside content, including two major‐effect QTLs controlling rebaudioside A accumulation, offering valuable targets for molecular breeding of high‐quality stevia cultivars (Sharma et al. 2024).

### 4.3 Marker‐Assisted Selection (MAS) Breeding

Marker‐assisted selection has generated numerous successful commercial and near‐commercial applications in sugar crops, with particularly pronounced impacts on the improvement of disease resistance. In sugarcane, the identification of the brown‐rust resistance gene *Bru1* and the development of tightly linked molecular markers have enabled widespread implementation of MAS across major production regions. These markers allow breeding programmes to rapidly screen germplasm for *Bru1* resistance alleles and to efficiently introgress them into high‐sugar, high‐yielding parental backgrounds, thereby substantially reducing yield losses caused by brown rust (Aljanabi et al. 2007; Costet et al. 2012; Glynn et al. 2013).

In sugar beet, MAS‐based resistance gene pyramiding represents one of the most successful breeding paradigms. The use of tightly linked or functional markers for the *rhizomania* resistance genes *Rz1* and *Rz2* has enabled breeders to efficiently stack these loci within elite genetic backgrounds, resulting in cultivars with broad‐spectrum and durable resistance to beet necrotic yellow vein virus and markedly improved yield stability in affected production areas (Gidner et al. 2005). Similarly, markers linked to the nematode‐resistance gene *Hs1pro‐1* have accelerated the development of sugar beet cultivars resistant to beet cyst nematode.

Beyond disease resistance, MAS has also been applied to improve quality‐related and developmental traits in sugar beet. For example, analysis of 126 Chinese sugar beet breeding lines characterized the distribution of fertility‐related genes and genotyped both cytoplasmic and nuclear fertility factors, providing a genetic foundation for hybrid‐breeding programmes (Peng et al. 2023). In addition, QTL‐linked markers associated with reduced impurity contents (potassium and sodium) and favourable root‐shape traits have been incorporated into selection schemes to improve processing quality and mechanized harvestability. For bolting resistance, functional markers linked to allelic variation in *BvFL1* have even been used to screen parental lines and breeding materials with stronger vernalization requirements and a reduced risk of premature flowering.

### 4.4 Genomic Selection Shows Strong Promise for Predicting Complex Traits

Regarding complex quantitative traits governed by numerous small effect loci—such as cane yield, sugar content and biomass—the efficiency of marker‐assisted selection is inherently limited (Robarts and Wolfe 2014). Genomic selection (GS) represents a transformative breeding strategy that exploits genome‐wide marker information, typically tens to hundreds of thousands of SNPs, to construct predictive models that estimate the breeding value of an individual without the need to pre‐identify specific genes or quantitative trait loci (Meuwissen et al. 2001; Crossa et al. 2017). By shifting selection decisions from retrospective evaluation to prospective prediction, which is based on prolonged field trials and genome‐wide information respectively, GS can substantially reduce the length of the breeding cycle and improve selection accuracy, particularly for low‐heritability traits (Lee 1995).

In sugarcane, genome‐wide marker‐based genomic selection is widely regarded as a key approach to partially replace extensive field phenotyping with predictive modelling. Prediction models are trained using a reference population with both genotypic and phenotypic data and then applied to un‐phenotyped materials, enabling earlier selection decisions and substantially shortening breeding cycles (Mahadevaiah et al. 2021; Yadav et al. 2020). Early proof‐of‐concept studies using two commercial breeding panels from Réunion Island and Guadeloupe (167 clones each, 334 total) genotyped with 1499 DArT markers demonstrated the feasibility of genomic prediction for ten agronomic traits in sugarcane (Gouy et al. 2013). Subsequent analysis further assessed genomic prediction accuracy for cane yield and recoverable sugar traits, including commercially relevant indices such as CCS (Deomano et al. 2020). More recent quantitative results from the Louisiana sugarcane breeding programme, using 95 commercial clones genotyped with 3906 SNPs, reported prediction accuracies (rank correlation) of 0.47–0.80 for sucrose content, 0.61–0.69 for cane yield and 0.56–0.72 for sugar yield (Satpathy et al. 2022). At Cenicaña (Colombia), genomic prediction models achieved accuracies of 0.64 for sucrose, 0.63 for stem diameter and 0.59 for waterlogging tolerance.

In addition to yield related traits, genomic selection has been evaluated for predicting resistance to brown rust and orange rust (Islam et al. 2021). More application‐oriented studies have exploited historical ordinal or classified disease response data to compare the performance of non‐parametric and machine‐learning models, including support vector machines, random forests and neural networks, in predicting resistance to multiple sugarcane diseases (O'Connell et al. 2022; Chen, Bhuiyan, et al. 2024; Shahi et al. 2026). These studies collectively indicate that GS can achieve moderate to high predictive ability for complex traits in sugarcane, with Bayesian models generally outperforming rrBLUP (Satpathy et al. 2022).

Overall, genomic selection has demonstrated strong potential across sugar crops by enabling substantial reductions in breeding‐cycle length and by improving selection accuracy for low‐heritability traits such as yield (Lee 1995). In sugar beet, which has a well‐characterized diploid genome, GS has advanced more rapidly. In a large population of 924 lines, cross‐validation correlations > 0.8 between observed and predicted testcross performance were observed for sugar content (Würschum et al. 2013). Several leading sugar beet breeding programmes have integrated GS into commercial pipelines, achieving prediction accuracies for root yield and sugar content comparable to or even exceeding those obtained through conventional phenotypic selection. Machine‐learning approaches, such as random forests, have further enhanced predictive performance, achieving 98.5% accuracy in distinguishing modern breeding lines from public seed bank accessions (Sandell et al. 2025). In sweet sorghum, GS has been evaluated for agronomic performance under contrasting environmental conditions, including drought and irrigated treatments, with predictive models achieving moderate accuracies for biomass‐ and sugar‐related traits. For stevia, GS is still at the early stages, with most efforts focused on QTL mapping for rebaudioside A and stevioside content (Sharma et al. 2024).

Despite these advances, several challenges remain, particularly for polyploid crops such as sugarcane. Accurately incorporating allelic dosage information and complex dominance and epistatic effects into genomic prediction models remains a major frontier in statistical genetics and computational biology. Predictive performance is strongly influenced by the size and diversity of the training population and also by its genetic relatedness to the target breeding population, yet establishing and maintaining large, high‐quality training populations entails substantial costs. In addition, marker effects often vary across environments, highlighting the need for genomic selection models that can integrate environmental covariates or support multi‐environment analyses to facilitate breeding for broad adaptation (Xu et al. 2022). Although sequencing costs continue to decline, genome‐wide genotyping of large breeding populations still represents a significant investment. Optimizing sequencing strategies and genotype‐imputation methods will therefore be critical for scaling up genomic selection in practical sugar crop breeding programmes.

The integration of deep learning and artificial intelligence is opening new frontiers for GS. Novel deep‐learning frameworks—such as CropARNet, which integrates a self‐attention mechanism with a deep residual network—have been benchmarked on 53 key agronomic traits across rice, maize, cotton and millet, ranking first in prediction accuracy for 29 traits (Zhou et al. 2025). Similarly, ResDeepGS has improved prediction accuracy by 5%–9% on wheat datasets (Yan et al. 2025). Most recently, the integrated genomic‐environmic prediction (iGEP) framework has been proposed as an extension of GS that treats environmental components with comparable dimensionality to genotypes and phenotypes, substantially improving prediction accuracy under strong genotype by environment interactions (Xu et al. 2022). For sugarcane, an AI‐driven breeding roadmap centred on iGEP has been systematically outlined, offering a theoretical framework for efficient improvement of complex genome crops (Wang, Zhang, Zou, et al. 2026). As genotyping costs continue to decline and multi‐environment phenotyping datasets accumulate, the convergence of GS with AI and environmic information will accelerate genetic gain and shorten breeding cycles across all sugar crops. For quick reference, Table S3 summarizes the major types of molecular markers, their characteristics and representative applications in sugar crop breeding.

## 5 Genetic Transformation and Genome‐Editing

Genetic transformation enables the introduction of exogenous genes into plant cells to generate novel traits, whereas genome editing allows precise modification of endogenous genomic sequences. Together, these complementary technologies constitute the engineering core of modern crop genetic improvement and provide unprecedented precision for trait manipulation in sugar crops.

### 5.1 Increasingly Optimized Transformation Systems

Transformation efficiency varies widely among sugar crops and is largely determined by their capacity for tissue culture, regeneration, and susceptibility to gene delivery. As a monocotyledonous poaceae crop, sugarcane has historically posed major challenges for genetic transformation. Key bottlenecks include strong genotype dependence in regeneration systems and relatively low efficiency of *Agrobacterium tumefaciens* –mediated infection. Consequently, particle bombardment was initially the most widely used method; however, this approach is costly and frequently results in high transgene copy numbers and transgene silencing (Elliott et al. 1998; Manickavasagam et al. 2004; Meng et al. 2023). In recent years, substantial improvements have been achieved in *Agrobacterium*‐mediated transformation of sugarcane through optimization of *bacteri*al strains—such as the use of hypervirulent strain EHA105—co‐delivery of helper proteins (e.g., *P19* and virulence genes), selection of highly responsive explants (including young leaf‐base meristematic tissues) and refinement of culture media and regeneration conditions. Collectively, these advances have increased transformation efficiency and promoted the recovery of low‐copy, stably inherited transgenic events (Mayavan et al. 2015).

More recently, transformation strategies based on developmental regulators (DRs) have progressed rapidly. These approaches aim to enhance regeneration competence during transformation by modulating developmental programmes through hormone balance and nutrient availability, thereby improving plant recovery following gene delivery. In recent years, DR‐based systems have attracted considerable attention and intensive investigation (Wang, Si, et al. 2025), as multiple regulators have been shown to markedly improve tissue‐culture regeneration across diverse plant species (Debernardi et al. 2020; McFarland et al. 2023; Xu et al. 2025). Notably, co‐expression of the AP2/ERF transcription factor BABY BOOM (bbm) and the shoot apical meristem regulator WUSCHEL2 (wus2) significantly enhances callus transformation efficiency in sorghum, sugarcane, indica rice and even previously recalcitrant maize inbred lines (Lowe et al. 2016). Building on this concept, a recent study reported a novel, highly efficient and largely genotype‐independent transformation system for sugarcane. A key innovation was the identification of a specific meristematic region with exceptionally high regenerative capacity as the explant, combined with customized *Agrobacterium* strains, vector designs and optimized culture workflows. This system enabled stable, high‐frequency regeneration of transgenic plants across multiple sugarcane genotypes, including major commercial cultivars, with efficiencies far exceeding those of earlier methods (Wang, Wang, Zhao, et al. 2026). This breakthrough effectively addresses a long‐standing bottleneck in sugarcane transformation and provides a robust technical foundation for large‐scale functional genomics and precision genetic improvement.

In contrast to sugarcane, sugar beet, as a dicotyledonous species, possesses one of the most mature and efficient transformation systems among sugar crops. Explants such as cotyledons and hypocotyls are highly susceptible to *Agrobacterium tumefaciens* , and standard *Agrobacterium*‐mediated protocols typically achieve high transformation and regeneration efficiencies. Transgenic sugar beet was first reported in 1987, and since then transformation has been widely applied to improve disease resistance, herbicide tolerance and carbohydrate composition (Ehlers et al. 1991; Mannerlöf et al. 1996; Wright and Penner 1998; Zhuzhzhalova et al. 2019).

In *S. rebaudiana* , genetic transformation primarily relies on *Agrobacterium*‐mediated leaf‐ or stem‐segment systems, which are relatively well established and have been extensively used for metabolic engineering of steviol glycosides. In sweet sorghum, transformation approaches largely follow established poaceae models such as maize and rice, with both *Agrobacterium*‐mediated and biolistic methods being applied, although transformation efficiency remains strongly genotype‐dependent (Li, Pan, et al. 2024). For woody sugar crops, including palms and sugar maple, transformation systems are still at an exploratory stage and regeneration recalcitrance remains the primary barrier to efficient genetic modification.

### 5.2 Rapidly Expanding Landscape of Transgenic Approaches

The extremely high ploidy level and pronounced heterozygosity of sugarcane substantially limit the efficiency of trait introgression and pyramiding through conventional crossing and backcrossing. It has long constrained the rapid improvement of key traits such as insect resistance and herbicide tolerance (Shibao et al. 2021; Meng et al. 2026). In this context, the core advantages of transgenesis—namely ‘single introduction with long‐term expression’—together with the capacity of genome editing to precisely modify target loci and enable multi‐locus stacking, have driven sugarcane research beyond single‐gene proof‐of‐concept studies toward more systematic engineering pipelines aimed at multi‐trait integration and regulatory‐compliant commercialization.

For lepidopteran pests such as the sugarcane borer, expression of *Bacillus thuringiensis* (Bt) toxins remains one of the most mature and widely validated engineering strategies. Demonstrating clear translational potential, simultaneous expression of two Bt proteins with distinct modes of action, *Cry1Ab* and *Cry2Ab*, in a commercial sugarcane background provided effective field‐level protection against sugarcane borers (Cristofoletti et al. 2018). Similarly, transgenic sugarcane expressing *cry1Ac* exhibited enhanced resistance to borer infestation and improved overall phenotypic performance, accompanied by elevated expression of endogenous stress‐response genes associated with borer attack (Gao et al. 2016; Zhou et al. 2018). Using particle bombardment, an expression cassette containing *cry2A* was introduced into the widely cultivated Chinese variety ROC22, with bar as a selectable marker. Subsequent molecular characterization, including copy‐number estimation (≤ 4 copies), together with comprehensive field evaluations, confirmed stable transgene integration and favourable agronomic performance (Gao et al. 2018).

Compared with transgenic approaches to herbicide tolerance—such as those based on overexpression of *EPSPS*—genome editing offers a more target‐specific alternative, with *acetolactate synthase* (ALS) representing a classical target. Using CRISPR/Cas9, Oz et al. (2021) achieved multi‐allelic targeted editing of *ALS* in highly polyploid sugarcane, successfully generating functional variants associated with herbicide tolerance and corresponding tolerant phenotypes. Beyond insect resistance alone, industrial deployment often prioritizes combined trait packages compatible with integrated chemical weed‐management systems. Accordingly, transgenic sugarcane lines stacking insect resistance and herbicide tolerance—such as *Cry1Ab* expression combined with *EPSPS*‐associated tolerance—have been developed and systematically characterized for transgene expression, phenotypic stability and agronomic performance (Wang, Yang, et al. 2017). In addition, overexpression of the *albicidin‐detoxifying gene albD* in sugarcane significantly enhanced resistance to sugarcane chlorotic disease. Transgenic lines exhibiting high *AlbD* activity in young stems could effectively protect against systemic pathogen multiplication, which precedes economically damaging disease symptoms (Zhang et al. 1999).

In sugar beet, major insect pests include foliar feeders such as cabbage armyworm (*Mamestra brassicae*) and root‐infesting maggots ( *Tetanops myopaeformis* ). Introduction of *cry1A(b)* or *cry1C* genes via indirect gene transfer generated two transgenic sugar beet lines, NK150 and TK80, which exhibited effective resistance to foliar infestations by cabbage armyworm (Gurel et al. 2008). Beyond Bt toxins, additional resistance genes have been explored. For example, ipt genes encoding cytokinin biosynthesis enzymes have been shown to influence plant growth and defence responses (Smigocki et al. 2008). Transgenic hairy‐root lines expressing BvSTI, which encodes a serine (trypsin) protease inhibitor, displayed resistance to root maggot infestation (Smigocki et al. 2006). Moreover, genes such as *cry2A*, *cry1C*, *cp4*‐*epsps*, *gox*, *cry1A105* and *cryIIIAa* have also been evaluated in sugar beet insect‐resistance studies (Litvin et al. 2014; Moazami‐Goodarzi et al. 2020). Transgenic approaches have also been applied to enhance viral disease resistance in sugar beet. For beet necrotic yellow vein virus (BNYVV), resistance has been engineered using cross‐protection strategies involving expression of viral coat‐protein genes (Gurel et al. 2008). Similarly, insertion of antisense sequences derived from pathogen replicase genes into sugar beet cell lines enhanced antipathogen responses in regenerated plants, further demonstrating the feasibility of transgenic strategies for viral disease control (Lennefors et al. 2008; Vaz 2019).

### 5.3 Transformative Impact of Genome Editing

Sugarcane is an important energy crop, and genome engineering has provided powerful tools to improve biomass conversion efficiency. Early functional studies demonstrated that RNA interference–mediated suppression of the lignin‐biosynthesis gene *caffeic acid O‐methyltransferase* (*COMT*) can enhance bioethanol production from lignocellulosic biomass. Building on this concept, transcription activator‐like effector nucleases (TALENs) were used to introduce multi‐allelic mutations within conserved regions of *COMT*, thereby modifying lignin biosynthesis in sugarcane. Field‐grown TALEN‐mediated COMT mutants exhibited lignin reductions of up to 19.7%, that is direct evidence that targeted genome modification can improve bioenergy‐related traits in sugarcane (Kannan et al. 2018).

The emergence of the CRISPR/Cas system has further revolutionized precision breeding by enabling targeted editing of endogenous genomic sequences. Unlike transgenesis, which typically involves random integration of exogenous DNA, CRISPR‐based editing allows precise modification of native alleles and can generate edited plants that are difficult to distinguish from those produced by conventional mutagenesis or natural variation, offering potential advantages for regulatory approval and public acceptance. However, due to its extreme polyploidy, implementation of CRISPR/Cas technology presents unique challenges in sugarcane, as effective phenotypic modification often requires simultaneous disruption of multiple homoeologous alleles. By optimizing single‐guide RNA design to target highly conserved regions, driving Cas9 expression with strong promoters, and employing multiplex editing strategies, researchers have successfully achieved targeted gene knockouts in multiple sugarcane cultivars (Guo et al. 2024; Brant et al. 2024). In parallel, emerging editing modalities, including base editors and prime editors, are beginning to be explored in sugarcane, opening new possibilities for precise allelic replacement and single‐nucleotide substitutions.

Targeted knockout of susceptibility alleles represents a promising strategy for improving disease resistance. In sugarcane, CRISPR/Cas9 can be used to disrupt specific alleles of smut susceptibility–associated NBS‐LRR genes, with the aim of generating resistant germplasm. Similar strategies are being pursued in sugar beet, where editing of susceptibility genes is an active avenue for enhancing resistance to rhizomania and other diseases. In addition to disease resistance, genome editing offers opportunities to improve sugar accumulation. For example, knocking out or attenuating genes involved in sucrose catabolism, such as vacuolar invertases, could theoretically reduce sucrose breakdown in storage tissues and increase final sugar content (Wang, Zheng, et al. 2017; Shivalingamurthy et al. 2018).

Genome editing has also enabled precise manipulation of plant architecture to enhance light interception and biomass productivity. The grass‐specific gene LIGULELESS1 (LG1), which regulates ligule and auricle development, has been extensively edited in sugarcane, with up to 40 *LG1* alleles disrupted. One edited line, L35, exhibited a 56% reduction in leaf inclination angle, a 31% increase in tiller number, and a 25% increase in internode number, demonstrating the potential of multi‐allelic editing to optimize canopy architecture in polyploid crops (Brant et al. 2024). In sugarcane, genome editing of flowering‐promoting genes has also been explored as a strategy to delay or suppress flowering, thereby minimizing sugar loss associated with reproductive transition and reducing interference with breeding operations (Jayça et al. 2022; Pavani et al. 2023). In sugar beet, precise editing of cis‐regulatory elements in bolting control genes such as *BvFL1* offers the possibility of fine‐tuning vernalization requirements without adversely affecting other agronomic traits, facilitating the development of cultivars with broader environmental adaptation (Frerichmann et al. 2013). In addition, targeted disruption of genes essential for anther or pollen development provides an efficient route to generate male‐sterile lines, which is particularly valuable for hybrid‐seed production in crops such as sweet sorghum.

Despite these rapid advances, the application of genome editing in sugar crops continues to face challenges related to transformation and delivery efficiency, potential off‐target effects, the complexity of editing polyploid genomes and the lack of globally unified regulatory frameworks. Nevertheless, the exceptional versatility and precision of genome‐editing technologies are accelerating progress across sugar‐crop research and firmly positioning genome editing as a central driver of future precision breeding and molecular design strategies.

## 6 Future Perspectives

Biotechnology and molecular breeding have provided unprecedented tools and opportunities for sugar‐crop improvement. However, translating these advances into broad, stable and reproducible breeding gains remains challenging at both the scientific and technology‐integration levels. Overcoming these bottlenecks—and fully leveraging emerging approaches such as synthetic biology and artificial intelligence to enable deep cross‐disciplinary integration—will be essential to unlock the full potential of modern breeding technologies and to meet future demands for global food security and a sustainable bioeconomy.

### 6.1 Synthetic Biology Enables Metabolic Pathway Design for Coordinated Multi‐Trait Improvement

Beyond the manipulation of individual genes or single traits, synthetic biology and metabolic engineering aim to redesign plant metabolic networks in a systematic, predictable, and coordinated manner. This emerging frontier is transforming sugar crops from traditional agricultural commodities into programmable ‘green cell factories’, thereby opening new possibilities for sustainable production, climate resilience and high‐value biomanufacturing.

**FIGURE 5:**
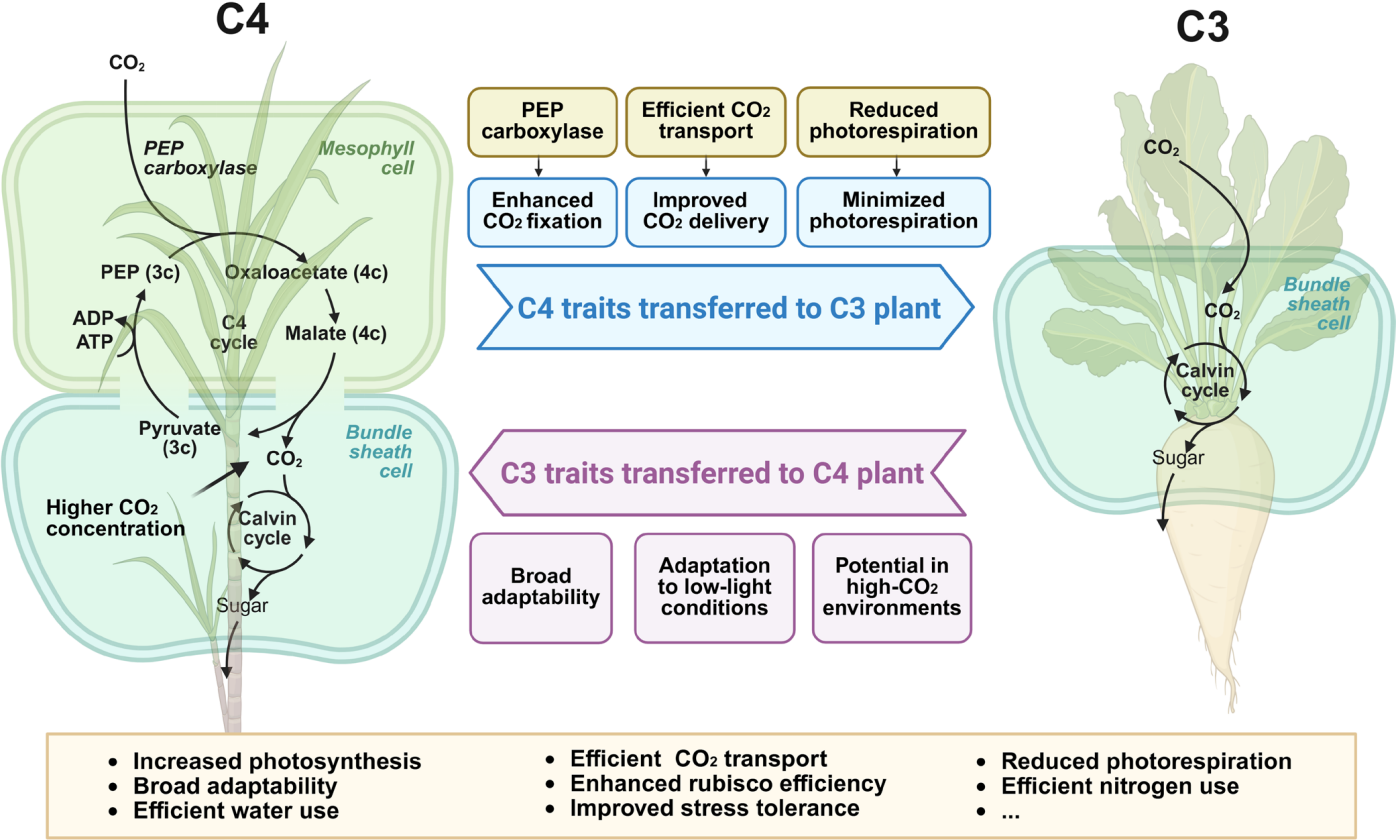
Photosynthetic pathways in C4 and C3 plants and their key characteristics. Schematic comparison of carbon fixation mechanisms in C4 and C3 plants, highlighting their distinct physiological features. The diagram emphasizes how insights from each pathway may be leveraged to complement respective strengths and mitigate limitations, enabling further improvement in photosynthetic efficiency. Created with BioRender (http://www.BioRender.com).

C3 and C4 plants have evolved distinct photosynthetic strategies adapted to different environmental conditions. As illustrated in Figure 5, C3 plants are broadly adapted ‘generalists’ under mild, humid conditions, whereas C4 plants such as sugarcane represent ‘specialists’ optimized for hot and dry environments with high water‐use efficiency. Even in C4 crops, further optimization remains possible under future climate scenarios characterized by elevated temperatures and water limitation (Jiang et al. 2023; Hua, Li, et al. 2024; Hua, Shi, et al. 2024). Synthetic biology increasingly offers the possibility of integrating advantageous features from both photosynthetic types into a single crop system. Notably, the oxygenase activity of Rubisco can still lead to inefficient photorespiration. Drawing on successful strategies developed in C3 species, introducing more efficient algal or bacterial photorespiratory bypass pathways, such as the glycolate oxidase‐catalase (GOC) bypass, into sugarcane or sweet sorghum may further reduce carbon loss and energy consumption (Zhao et al. 2025).

For the C3 crop sugar beet, engineering a functional C4‐like photosynthetic system represents a far more complex, systems level challenge, requiring modifications in leaf anatomy, establishment of bundle sheath‐like cellular specialization and precise cell‐type‐specific expression of key enzymes (Schuler et al. 2016). Despite these challenges, accumulating evidence suggests that implementing elements of C4 metabolism in C3 plants is a feasible long‐term objective. For example, the introduction of maize genes encoding multiple C4 enzymes into rice resulted in a tenfold increase in PEPC expression, indicating that coordinated upregulation of carbon‐metabolism enzymes can establish partial C4‐like functionality (Ling et al. 2006; Swift et al. 2024; Ermakova et al. 2021). Nonetheless, substantial challenges remain, including optimization of both biochemical fluxes and anatomical traits, and assessing the need for transporter engineering to accelerate metabolite exchange between mesophyll and bundle sheath compartments.

Synthetic biology also provides new strategies to systematically re‐engineer carbon flux across the entire ‘source‐flow‐sink’ continuum. Enhancing ‘flow’ efficiency, specifically phloem loading and long‐distance transport, can be pursued through rational design of SUT expression patterns, subcellular localization and transport capacity (Zhu, Lan, Zhao, et al. 2025). At the sink level, potential strategies include downregulating or knocking out key enzymes involved in sucrose degradation (e.g., vacuolar invertase and the sucrose‐cleavage activity of sucrose synthase), strengthening vacuolar sucrose sequestration through engineering of tonoplast sucrose transporters and coordinately modulating phytohormone pathways that control cell division and expansion to increase both the number and volume of storage cells (Verma et al. 2019; Mehdi et al. 2024). Although most synthetic‐biology applications remain at the proof‐of‐concept stage in sugar crops and face challenges such as metabolic flux balancing and trade‐offs among growth, defence and productivity, this approach holds the potential to shift breeding paradigms from ‘domestication by selection’ toward ‘rational metabolic design’ (Sedelnikova et al. 2018; Swift et al. 2024).

### 6.2 An Integrated Breeding Platform Enabled by Multi‐Technology Convergence

Future breakthroughs in sugar‐crop improvement will depend on the establishment of a new integrated breeding paradigm that is driven by multi‐omics data, enabled by intelligent decision‐making, and implemented through a closed‐loop, iterative process (Wang, Wang, et al. 2024, 2025g; Zhu, Li, et al. 2025). This paradigm does not represent a simple linear stacking of technologies; rather, it constitutes a dynamic and cyclic system composed of tightly interconnected layers.

At the data‐integration layer, genomics, transcriptomics, metabolomics and high‐throughput phenomics data are systematically integrated to construct ultra‐high‐dimensional genotype–phenotype association networks, enabling comprehensive dissection of the biological mechanisms underlying trait formation. At the knowledge‐mining layer, artificial intelligence and machine‐learning approaches are applied to these integrated datasets to identify key regulatory genes, favourable haplotypes, high‐efficiency molecular markers and predictive biomarkers that are most relevant for breeding objectives (Feng et al. 2024). At the operational layer, efficient and precise genetic manipulation tools—including optimized transformation systems and multiplex genome‐editing technologies—are used to rapidly introduce, modify or recreate the target alleles identified through data mining within elite genetic backgrounds. Finally, at the evaluation and prediction layer, newly generated germplasm is subjected to high‐throughput, multi‐environment phenotyping and the resulting data are continuously fed back into genomic selection models and AI algorithms. This iterative feedback enables ongoing optimization of parental selection, early‐generation screening and prediction of cultivar performance across diverse environments, thereby guiding successive rounds of breeding design (Zhu, Li, et al. 2025).

Within this integrated framework, digital twins and trusted data infrastructures are expected to evolve from auxiliary analytical tools into core breeding infrastructure. Digital twin systems enable pre‐testing of cultivar performance and management strategies in virtual environments, reducing experimental cost and risk. At the same time, blockchain‐enabled human‐cyber‐physical system architectures can enhance traceability of multi‐source data, improve the credibility of trusted data sharing, and strengthen cross‐entity collaboration, thereby supporting large‐scale joint breeding programmes and precision agriculture decision‐making. In parallel, advances in single‐cell and spatial omics are expanding the resolution of breeding targets from the tissue level to individual cell types, providing unprecedented opportunities for cell type‐directed regulation of carbon allocation and metabolic activity (Zhu, Li, et al. 2025).

In the context of accelerating climate change, future sugar crop cultivars must exhibit ‘smart’ adaptability (Zytynska 2021). This includes the development of broadly adapted cultivars with reduced sensitivity to photoperiod and temperature, cultivars with dynamic stress response capacities (e.g., rapid stomatal closure under drought and efficient growth recovery when resources become available), and cultivars with enhanced carbon‐sequestration potential achieved through synthetic biology approaches. The deep integration of biotechnology, molecular breeding and digital technologies is steadily moving the field toward an era in which climate‐smart elite cultivars can be rationally designed and efficiently created.

## 7 Conclusion

Obviously, traditional breeding approaches, constrained by long selection cycles and limited capacity to manipulate complex traits, are increasingly inadequate to meet modern demands for higher yield, greater efficiency, enhanced resilience and diversified end uses. In the context of escalating global challenges—including climate change, food security and the transition toward a sustainable bioeconomy—the genetic improvement of sugar crops is undergoing a profound paradigm shift driven by advances in biotechnology and molecular breeding. This review has systematically illustrated how cutting‐edge technologies—including genomics, molecular marker–assisted selection, genomic selection, genome editing and synthetic biology—are reshaping sugar‐crop breeding from an empirically driven process into a precision‐oriented, design‐based discipline. Collectively, these advances point toward a future in which interdisciplinary technological integration transforms sugar crops from conventional sugar‐producing plants into versatile, sustainable platforms for green biomanufacturing.

### 7.1 Paradigm Revolution in Genetic Improvement of Sugar Crop Through Traditional and Modern Approaches

Sugar‐crop improvement is shifting from experience‐driven decision‐making toward knowledge‐driven design, and from phenotype‐based selection toward genome‐informed engineering. This review has synthesized the major advances achieved over the past decade in sugar‐crop biotechnology and molecular breeding, highlighting that these developments represent not merely an accumulation of new tools but also a fundamental transformation of the breeding paradigm. The emergence of high‐quality reference genomes and multi‐omics resources has unlocked the genetic complexity of polyploid sugarcane and provided refined genetic blueprints for crops such as sugar beet and sweet sorghum, enabling trait dissection to progress from broad QTL intervals to precise identification of causal genes and allelic variants. The integration of molecular marker–assisted selection and genomic selection has further transformed breeding strategies by moving selection decisions from retrospective evaluation based on prolonged field trials to prospective prediction based on genome‐wide information. This transition has markedly improved selection efficiency and accuracy, particularly for complex quantitative traits and disease resistance. In parallel, breakthroughs in genetic transformation systems—especially the development of efficient and increasingly genotype‐independent protocols—have removed long‐standing technical barriers to functional genomics and genetic engineering in recalcitrant crops such as sugarcane. The widespread adoption of genome‐editing technologies, exemplified by CRISPR/Cas systems, has introduced an unprecedented level of precision, enabling the targeted creation of novel alleles and elite germplasm that are rare or absent in natural populations.

Looking forward, the rise of synthetic biology and metabolic engineering represents a conceptual leap beyond incremental trait improvement. By re‐envisioning crops as programmable biological systems, these approaches signal a transition from simply ‘improving crops’ to rationally ‘designing crops’ with coordinated, multi‐trait optimization. Taken together, the convergence of these technologies has transformed sugar‐crop breeding from a largely empirical practice into a predictable, targeted and accelerated precision science. This paradigm shift provides a robust foundation for developing climate‐resilient, high‐efficiency and multifunctional sugar‐crop cultivars capable of supporting global sugar security, sustainable bioeconomic growth and long‐term environmental resilience.

### 7.2 Only Path to Achieve the Next Breakthrough in Breeding Through Interdisciplinary Integration

Future challenges—including climate change adaptation, efficient resource utilization and the increasingly diverse demands of downstream industries—cannot be addressed through the linear or isolated application of individual technologies. Achieving the next major breakthrough in sugar‐crop breeding will depend on deep interdisciplinary collaboration and the systematic integration of cutting‐edge approaches across traditionally separate domains. The emerging breeding paradigm is best conceptualized as a ‘closed‐loop intelligent system’, in which discovery, implementation, evaluation and optimization are continuously linked. Within this system, data scientists play a central role by developing artificial intelligence algorithms capable of extracting latent patterns and predictive rules from vast, high‐dimensional multi‐omics and phenotypic datasets. Synthetic biologists and metabolic engineers are required to design, assemble and fine‐tune complex genetic circuits and metabolic networks that enable coordinated multi‐trait optimization. Engineers contribute by developing low‐cost, high‐throughput robotic platforms and intelligent phenotyping systems that can generate precise, scalable and reproducible trait measurements under diverse environments. In parallel, bioinformaticians are tasked with constructing digital twin models that simulate genotype–environment–management interactions, allowing virtual testing of breeding strategies and cultivar performance prior to field deployment.

Only through the seamless integration of genomics, phenomics, genome editing, data science and automation technologies—and by establishing a complete, iterative ‘design–create–test–learn’ cycle—can breeding systems achieve collaborative optimization of complex traits. Such integration is essential for accelerating the development of the next generation of high‐performance, climate‐resilient sugar crops and for transforming breeding from a sequential process into a continuously learning, self‐improving system capable of delivering breakthroughs at unprecedented speed.

### 7.3 A Bright Future for Sugar Crops as Adequate Sugar Sources and Sustainable Biomanufacturing Platforms

In the future, continued advances in biotechnology are poised to greatly expand both the traditional roles and the strategic value of sugar crops. These crops are no longer expected to function solely as primary producers of sugar and ethanol; rather, they are increasingly evolving into core platforms for sustainable biomanufacturing. Through targeted metabolic engineering, the inherently high biomass productivity and sugar content of sugar crops can be efficiently redirected toward the production of biobased materials, specialty chemicals, pharmaceutical intermediates and even recombinant proteins, thereby embedding sugar crops more deeply within an expanded green bioeconomy value chain.

At the same time, ‘climate‐smart’ cultivars developed through molecular design and precision breeding will exhibit enhanced tolerance to abiotic stresses, enabling stable productivity on marginal lands and strengthening resilience in global food and energy systems. Such advances will be particularly important under future climate scenarios characterized by increasing environmental variability and resource constraints. Ultimately, through global cooperation and open innovation frameworks, these technological breakthroughs are expected to be more widely shared and equitably applied, promoting sustainability and fairness across the global agricultural system. Continued investment and innovation in sugar‐crop biotechnology and molecular breeding are therefore essential for the long‐term vitality of the sugar industry, representing a critical cornerstone for building a more sustainable, resilient and inclusive bio‐economic society.

## Author Contributions

Conceptualization: WP, WY, and QY; Writing original draft preparation: WP; Review and editing: WP, WQ, WW, LP, LY, MK, WY, and QY; Funding acquisition: QY. All authors have read and agreed to the published version of the manuscript. All authors read and approved the final manuscript.

## Funding

This work was funded by Chinese Academy of Tropical Agricultural Sciences for Science and Technology Innovation Team of National Tropical Agricultural Science Center (CATASCXTD202402), Guangxi Science and Technology Project (Agricultural and Rural Field) (GUIKENONG AB24153001 and GUIKENONG AB24153007), Project of State Key Laboratory of Tropical Crop Breeding (NKLTCBCXTD38, NKLTCBCXTD24, and SKLTCBYWF202504), Central Public‐interest Scientific Institution Basal Research Fund (1630052022005), and China Agriculture Research System of MOF and MARA (CARS‐17).

## Consent

The authors have nothing to report.

## Conflicts of Interest

The authors declare no conflicts of interest.

## Supporting information


**Table S1:** Sources of sugar in plants.

**Table S2:** Major genes involved in key agronomic traits of sugar crops.

**Table S3:** Summary of molecular markers used in sugar crop breeding.

**Data S1:** pbi70683‐sup‐0004‐DataS1.docx.

## Data Availability

Data sharing not applicable to this article as no datasets were generated or analysed during the current study.
