# Supplementary material for: Revisiting the Molecular Roadmap for Sugar Crops: Genome Reading, Trait Writing and Variety Redesigning
Source: Plant Biotechnol J. 2026 May 13;24(9):5013–40. doi: 10.1111/pbi.70683 (PMC13399145; doi:10.1111/pbi.70683)
Supplement: Supplementary file 1 [file PBI-24-5013-s002.docx]

**Table.S1 Sources of sugar in plants**

| Sources | Common Name | Scientific Name |
| --- | --- | --- |
| Sucrose | Sugarcane | *Saccharum officinarum* |
|  | Sugar beet | *Beta vulgaris* |
|  | Sugar maple, Maple tree | *Acer saccharum* |
|  | Black maple, Black sugar maple, Hard maple, Rock maple | *Acer nigrum* |
|  | Sweet sorghum, sorgo | *Sorghum bicolor* |
|  | Palms (various names) | *Arecaceae Bercht. & J. Presl* |
| Glucose | Corn, maize (corn syrup or crystallized corn sugar from starch) | *Zea mays* |
|  | Potato, White potato (from starch) | *Solanum tuberosum* |
| Fructose | Dahlia (from inulin) | *Dahlia pinnata* |
|  | Jerusalem artichoke, sunchoke, sunroot (from inulin) | *Helianthus tuberosus* |
|  | Corn, maize (high-fructose corn syrup from starch) | *Zea mays* |
| Galactose | Flax, Linseed (from flaxseed gum or mucilage) | *Linum usitassitimum* |
| Mannose | Ivory nut, Ivory nut palm (from endosperm) | *Phytelephas macrocarpa* |
|  | Manna ash tree, Flowering ash (from juice secreted through the bark) | *Fraxinus ornus* |
| Maltose | Barley | *Hordeum vulgare* |
|  | Rice | *Oryza sativa* |

Table S2. Major genes involved in key agronomic traits of sugar crops

| **Crop** | **Trait category** | **Gene name** | **Function** |
| --- | --- | --- | --- |
| Sugarcane | Sugar metabolism | *SPS1, SPS2* | Sucrose-phosphate synthase, sucrose synthesis |
|  |  | *Sus1, Sus2 (SuSy)* | Sucrose synthase, sucrose cleavage in sink tissues |
|  |  | *SAI, NI* | Soluble acid invertase / neutral invertase, sucrose hydrolysis |
|  | Biotic stress | *Bru1* | Brown rust resistance (NBS-LRR) |
|  |  | *ScDIR5/7/11/40* | Dirigent proteins, lignin biosynthesis for mechanical barrier |
|  |  | *ScWRKY2, ScWRKY4, ScWRKY6* | Transcriptional regulation of defense signaling |
|  |  | *ScOPR1, ScOPR2* | Oxophytodienoate reductase, jasmonic acid pathway |
|  | Abiotic stress | *ScPYL61* | ABA receptor, drought tolerance via stomatal regulation |
|  |  | *ScCAX2/3/4* | Ca^2+^/H^+^ exchanger, involved in smut susceptibility |
|  | Development / Agronomic | *ScFT3* | Florigen, flowering induction |
|  |  | *ScGA20ox* | Gibberellin biosynthesis, vegetative growth promotion |
| Sugar beet | Sugar metabolism | *SPS, SPP* | Sucrose-phosphate synthase and phosphatase |
|  |  | *UDP-glucose 4-epimerase* | Mediates trade-off between sugar accumulation and cell wall biosynthesis |
|  | Biotic stress | *Rz1, Rz2* | Rhizomania (BNYVV) resistance (CC-NBS-LRR) |
|  |  | *Hs1pro-1* | Nematode resistance (beet cyst nematode) |
|  | Abiotic stress | *BvM14-cystatin* | Cysteine protease inhibitor, salt tolerance |
|  |  | *BvbZIP* | ABA signaling, salt stress response |
|  | Development / Agronomic | *BvFL1* | Flowering repressor (FT homolog), vernalization requirement |
|  |  | *BvBTC1* | Bolting time control |
| Sweet sorghum | Sugar metabolism | *SPS, SuSy, invertases* | Sucrose-phosphate synthase, sucrose synthesis |
|  | Development / Agronomic | *Dw1, Dw2, Dw3* | Plant height regulation (dwarfing) |
|  |  | *SbTB1* | Tiller number control |
|  |  | *Dry* | Stem juiciness / moisture content |
| Stevia | Steviol glycoside biosynthesis | *UGT76G1, UGT85C2, UGT91D2* | UDP-glycosyltransferases, final steps of steviol glycoside biosynthesis |
|  |  | *UGT74G1* | Glycosyltransferase for rebaudioside A formation |
|  | Regulatory | *WRKY11, MYB111, SCL3* | Transcription factors co-regulating steviol glycoside and phenolic pathways |

Table S3. Summary of molecular markers used in sugar crop breeding

| **Marker type** | **Characteristics** | **Main applications in sugar crops** |
| --- | --- | --- |
| SSR (Simple Sequence Repeat) | Codominant, highly polymorphic, reproducible but low throughput | Genetic diversity analysis, cultivar identification, early genetic mapping |
| SNP (Single Nucleotide Polymorphism) | Abundant, genome-wide, high-throughput, compatible with automated genotyping | High-density genetic mapping, QTL detection, GWAS, genomic selection |
| InDel (Insertion/Deletion) | Simple, cost-effective detection (gel-based) | Targeted genotyping, variety discrimination |
| SCoT (Start Codon Targeted) | Targets start codons, moderately polymorphic | Genetic diversity assessment |
| KASP (Kompetitive Allele-Specific PCR) | SNP-based, flexible, low-cost for small to medium scale | Marker-assisted selection, QTL validation |
| DArT (Diversity Arrays Technology) | High-throughput, reduced representation, no prior sequence needed | Genomic selection, genetic mapping |
